# Supplementary material for: Structural basis for the toxicity of Legionella pneumophila effector SidH
Source: Nat Commun. 2023 Nov 3;14:7068. doi: 10.1038/s41467-023-42683-8 (PMC10624908; doi:10.1038/s41467-023-42683-8)
Supplement: Supplementary file 1 — Supplementary Information [file 41467_2023_42683_MOESM1_ESM.pdf]

Figure S1- SidH FL co-elutes with bacterial EF-Tu

A) Size-exclusion chromatography (column: S200 10/300) of SidH FL. The peak fraction, labelled with green bar was loaded onto 10% SDS-PAGE (right panel). B) Single-molecule mass analysis of purified SidH FL using Mass-photometry shows that the sample has a major population (72%; 324 kDa) of monomer SidH FL (253 kDa) with an additional mass gain of ~70 kDa. Two other minor species are SidH FL alone (5%; 252 kDa) and EF-Tu alone (4%; 52 kDa). C) Peptide mass finger printing shows that tryptic digestion of unknown protein (left panel: SDS-PAGE) leads to peptides which matches to the bacterial EF-Tu with a sequence coverage of ~97% (right panel). These experiments were performed at least three independent times with similar results. Source data are provided as a Source Data file.

Figure S1

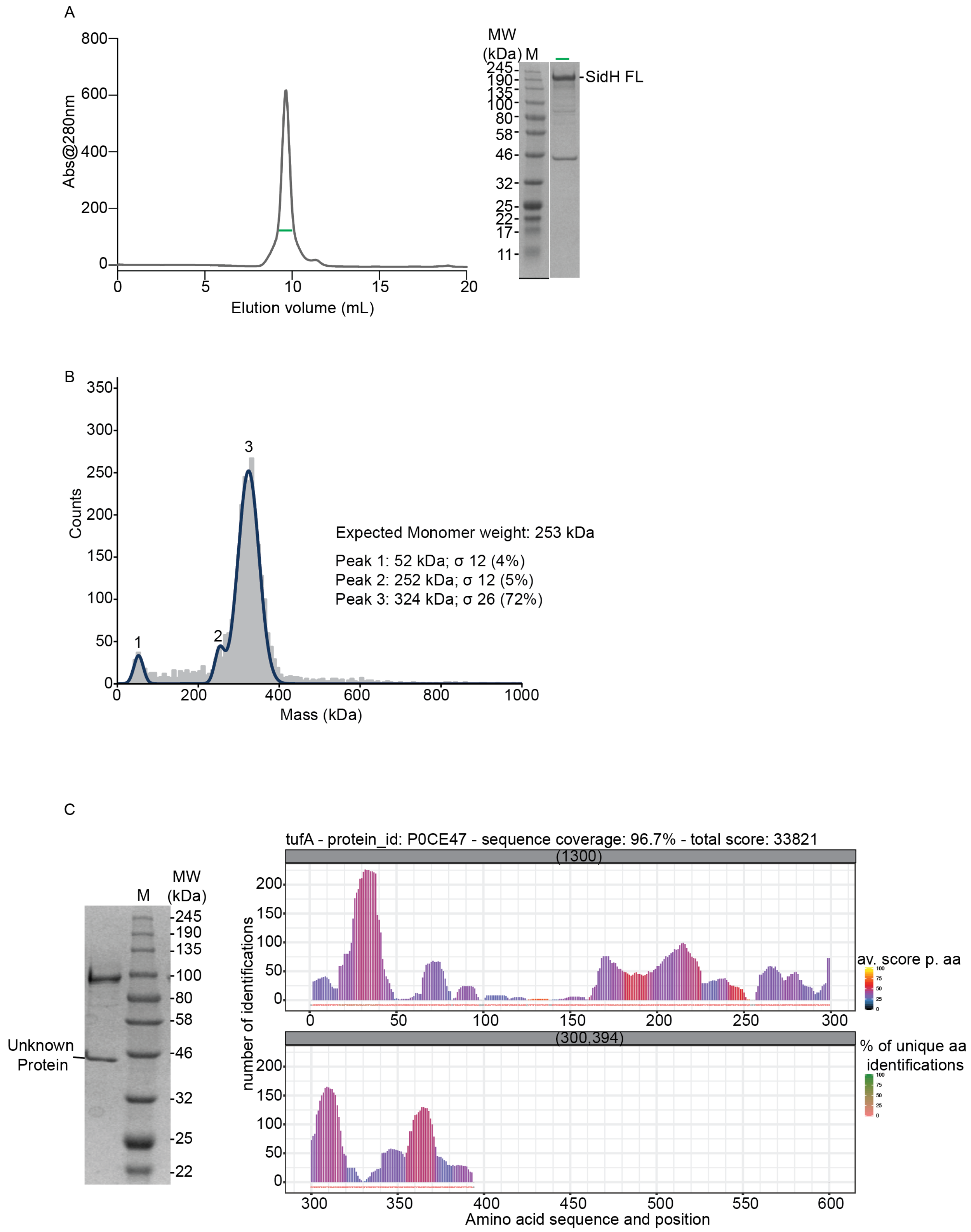
A
Abs@280nm
Elution volume (mL)
MW (kDa)
M
SidH FL
245
190
135
100
80
58
46
32
25
22
17
11
B
Counts
Mass (kDa)
Expected Monomer weight: 253 kDa
Peak 1: 52 kDa; σ 12 (4%)
Peak 2: 252 kDa; σ 12 (5%)
Peak 3: 324 kDa; σ 26 (72%)
C
Unknown Protein
M
MW (kDa)
tufA - protein_id: P0CE47 - sequence coverage: 96.7% - total score: 33821
(1300)
(300,394)
number of identifications
Amino acid sequence and position
av. score p. aa
% of unique aa identifications

Figure S2- cryo-EM image processing pipeline of SidH

Particles were picked using WARP and processed using RELION. The pipeline shows models upto post-processing step. Bottom left, Gold Standard FSC curves of SidH showing a final resolution of 2.7 Å.

Figure S2

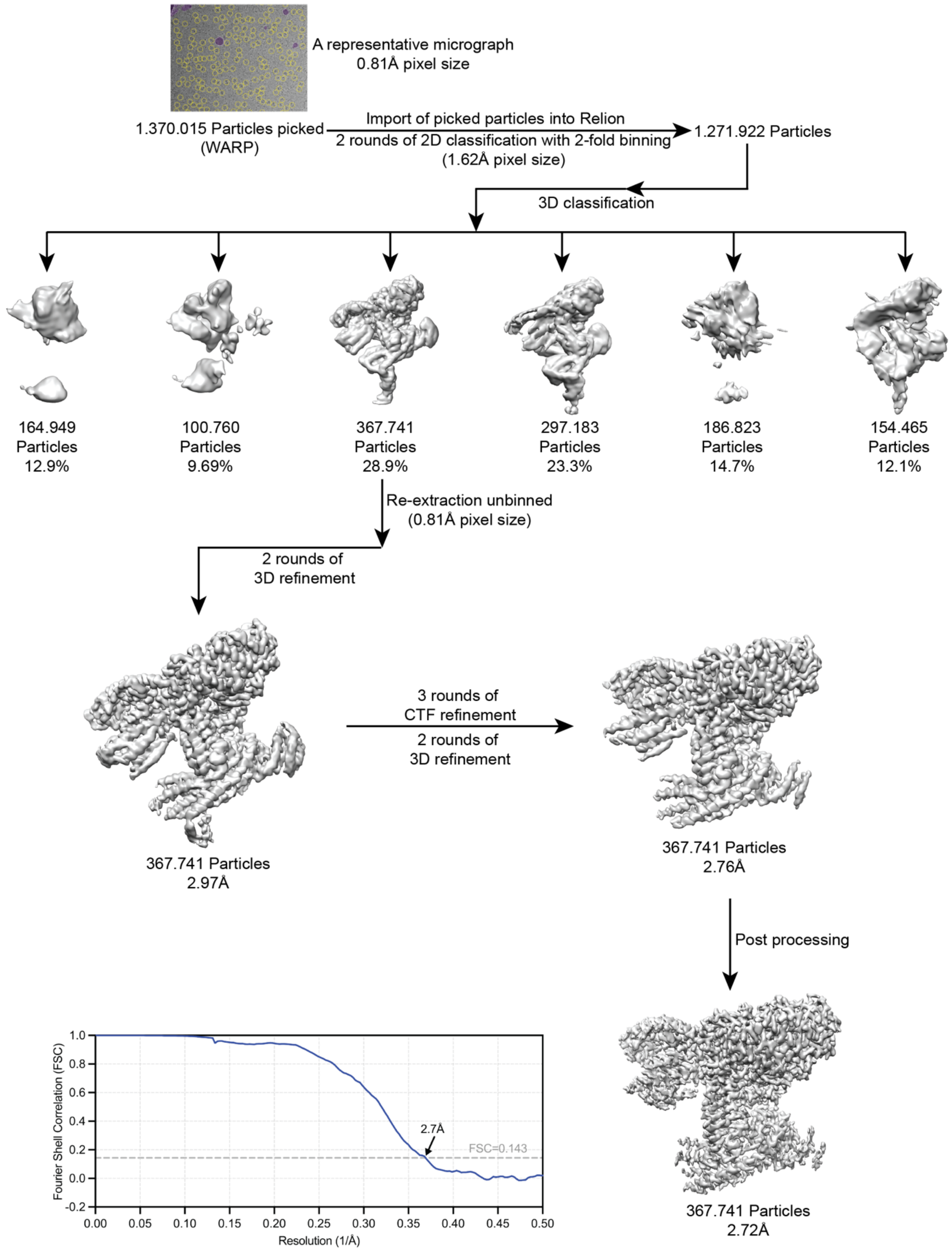
A representative micrograph
0.81Å pixel size
1.370.015 Particles picked
(WARP)
Import of picked particles into Relion
2 rounds of 2D classification with 2-fold binning
(1.62Å pixel size)
1.271.922 Particles
3D classification
164.949
Particles
12.9%
100.760
Particles
9.69%
367.741
Particles
28.9%
297.183
Particles
23.3%
186.823
Particles
14.7%
154.465
Particles
12.1%
Re-extraction unbinned
(0.81Å pixel size)
2 rounds of
3D refinement
367.741 Particles
2.97Å
3 rounds of
CTF refinement
2 rounds of
3D refinement
367.741 Particles
2.76Å
Post processing
367.741 Particles
2.72Å
Fourier Shell Correlation (FSC)
Resolution (1/Å)
2.7Å
FSC=0.143
1.0
0.8
0.6
0.4
0.2
0.0
-0.2
0.00
0.05
0.10
0.15
0.20
0.25
0.30
0.35
0.40
0.45
0.50

Figure S3- SidH structure showing EM density for tRNA
A) The "L" shaped double helical density is colored in pink. B) A $tRNA^{Phe}$ molecule from the X-Ray crystallographic structure (PDB: 1TTT) was used to fit into this density.

Figure S3

A

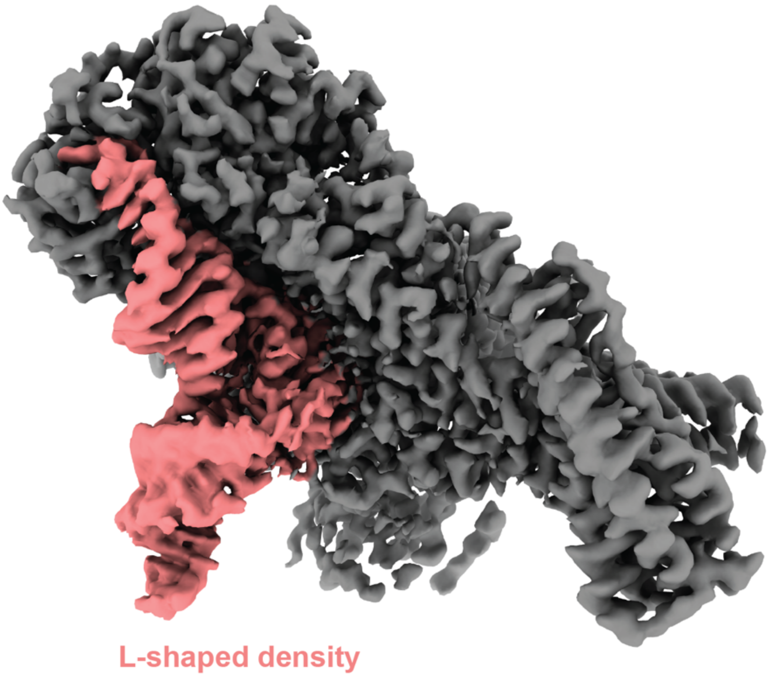

B

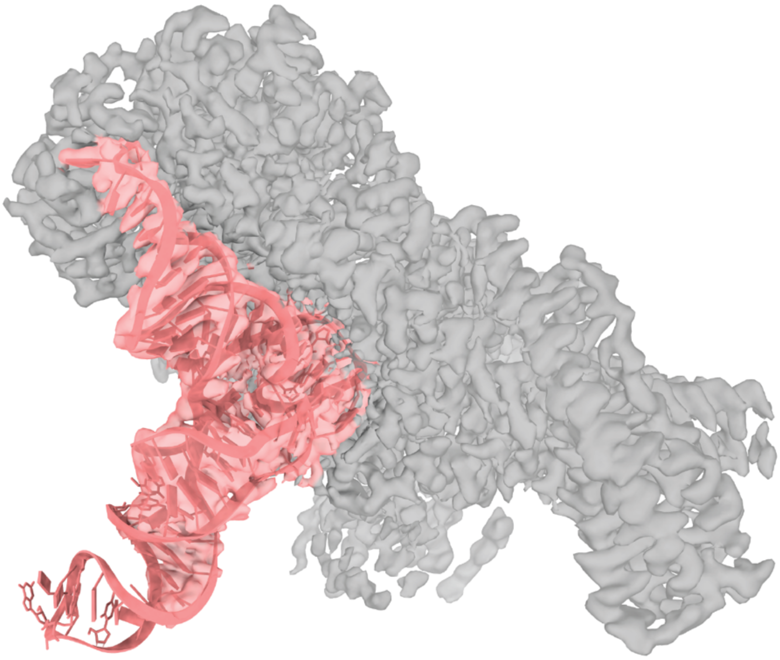

Figure S4- $tRNA^{Phe}$-EF-Tu model fitting in the density

A model of $tRNA^{Phe}$-EF-Tu from PDB: 1TTT was morphed into the density colored as green (EF-Tu) and pink ($tRNA^{Phe}$).

Figure S4

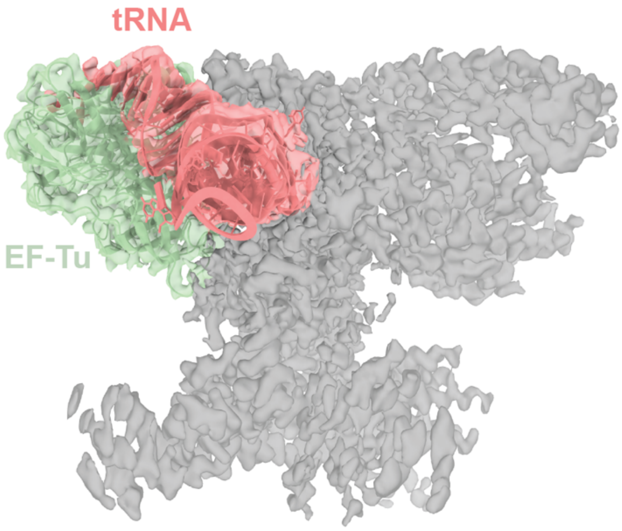

Figure S5- High sequence similarity between N- and C-terminal of SidH

Pairwise sequence alignment tool of EMBOSS Needle was used to predict the homology between N-term_SidH (1-615aa) and C-term_SidH (1028-1642aa).

Figure S5

```
N-term_SidH        1 MKRTIETYIIYLKEDLKIADTCKTIKDGLLKSITDKTHFSEELATYFERD 50
                                      :::.|    :||:.::..|   .|........:
C-term_SidH     1028 -----------------VSNFC----NGLVSNLPLK---QEPAPKVKAPE 1053

N-term_SidH       51 NPNAPFKVNTTDPTQVA----------------VLKKLLNALENAEKSFR 84
                     .|..|....||:|.:|.                :||::.:...:....::
C-term_SidH     1054 KPVTPVITGTTNPHEVVFGTKHEEFNSVYQPYLLLKRITDEFRDQNNPYK 1103

N-term_SidH       85 -------------------AIENIDISRDR-YTAMIAKDAVMVSYKAVHE 114
                                        .:|::|...|: :.|...:.:.::  ||:.|
C-term_SidH     1104 PSFDELKEEAVSYYDKIQPLLESVDPKFDKNFIAKHKESSTLL--KAIDE 1151

N-term_SidH      115 IYAALQLINHSNSDIQDIVGPHIQKLLPQMALASKALGNFAPEHPEESAG 164
                     :.:..|.||:..|....:...|::             |:|..|..:|
C-term_SidH     1152 VMSMRQRINNPESSFAKLKDLHLE-------------GDFEKEENKE--- 1185

N-term_SidH      165 AVLAGVVNMLPTEKPTESESLGKLSNLIFELPHYFEELQKLIATGASGIA 214
                                           |...|..|:..|   |.|:.:|......
C-term_SidH     1186 ----------------------KFRQLYKEIQPY---LHKIDSTYDQTQF 1210

N-term_SidH      215 TKSITSAEDYQSAMIKKANE------------------------------ 234
                     .:.:.:|:|:..|:.:..||
C-term_SidH     1211 LEGLKTAKDFSGALQRIMNEENALQQSTSLKDTSYLQLIAESLYQIPVKL 1260

N-term_SidH      235 --------------------TKYYFEQLSSKS-GLLAIPSYLSIVKRLIA 263
                                         .|.:.|.|:..| |..::...||...:|..
C-term_SidH     1261 NKLKAEPDTPEPSKEEIDANVKAFVEGLNGLSFGPGSVKKILSTAAKLQM 1310

N-term_SidH      264 HSTDLVNAGAPLTKQAYLDAVAKLEDIKHNILPQLISELEMVEESMGLKP 313
                     ..:|:...|..||       :.:|::|:......|::..:..|..:||||
C-term_SidH     1311 QLSDIGKEGRELT-------MGRLKEIQAEFGTILMAAADNAEFHLGLKP 1353

N-term_SidH      314 GLLTDPALEQMNKYYTQLAEQVDNIAKAAGVLDTVSDYSDSIGGKIVRFL 363
                     |..:....|:..|:|:.|   :.|:...          .|..|
C-term_SidH     1354 GTYSRTVSERFEKFYSSL---IVNLPLE----------KDQTG------- 1383

N-term_SidH      364 AGDSKKLDVGPKLTPAPDLGVLMDDVFIQKRRSNQESRLNEARLSSEDKS 413
                                       |.:|:|....|||.:.:..||...:   ||.|
C-term_SidH     1384 ------------------LELLIDTTSTQKRLAREMERLESVK---EDTS 1412

N-term_SidH      414 VLAAANRFF-DKIGSYNSIHKAWSKWSLANISQSEKDAL----------- 451
                     .:.......| .:...::::::.::  ||.:|::..:|.:
C-term_SidH     1413 AIDTKKSIFGTEHEQFSTLYQPYA--SLRHIAKDIEDGVHMNLYERTLEE 1460

N-term_SidH      452 -----IKEYKQFQPHFAALYPDIDKLVVDALTQPTGS----NIVSRLY-- 490
                          ..|||:.||:.|.:.|:..:   |.:::..|.    :.:.|::
C-term_SidH     1461 LKEEASNEYKKIQPYLAKINPEFTE---DYISKTDGEYSLLHAIDRVFEE 1507

N-term_SidH      491 -------------------SSDYKQLWSSDHFKQVLSCKDSVLSSI--QQ 519
                                        ..|:::..:.:.|.|:.:.....|..|  |.
C-term_SidH     1508 RHKINKPSSPFDKLRDLYLDGDFEKEENKEQFLQLYAELQPHLIKINYQY 1557

N-term_SidH      520 SLA--------QSEFKAKLIEKTMSHSEETAYSMNNKTTNLTTRVQPFEP 561
                     .||        ..:|||         :.|...:..:|...|.|.:...:.
C-term_SidH     1558 DLAYFLRELQTPEDFKA---------ATERIINDESKLQELITGLDDTKR 1598

N-term_SidH      562 LKFTLEDDKPVEYYHKRVIAASNQILEL--ERAQKGVAE-FFNYIQKKYP 608
                     ||..|.::: :.|:   :.....|.||:  |:.|....: |||||
C-term_SidH     1599 LKVKLCEER-IGYF---IDLLKKQELEVGPEKIQAFKEKIFFNYI----- 1639

N-term_SidH      609 HENPSFD 615
                     |.|
C-term_SidH     1640 HAN---- 1642
```

Figure S6- AlphaFold predicted model (HB6) fits into a part of EM density for DUF
A) Local resolution map showing poor electron density for Hb6, which is a part of DUF domain. B) AlphaFold predicted model was used to fit into the locally classified and refined density for Hb6.

Figure S6

A

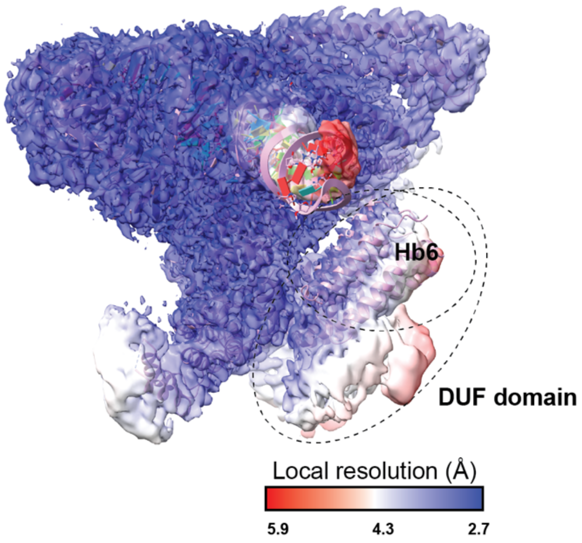
Hb6
DUF domain
Local resolution (Å)
5.9
4.3
2.7

B

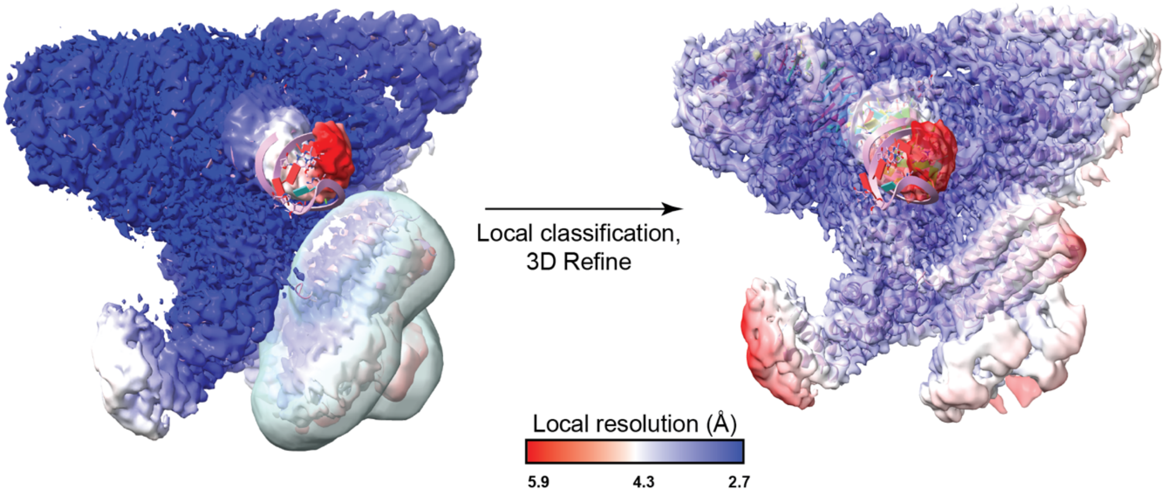
Local classification,
3D Refine
Local resolution (Å)
5.9
4.3
2.7

Figure S7- Structural similarity of SidH FL and expression of different SidH constructs in HEK cells

A) Structural alignment between SidH Hb6 (blue) and TSPO (translocator protein) (green) over 118 C- α s shows an rmsd of 3.2 Å. B) Western blot data showing the expression of various SidH proteins in HEK cells corresponding to the toxicity assay shown in Figure 1F. This experiment was performed at least three independent times with similar results. Source data are provided as a Source Data file.

Figure S7

A)

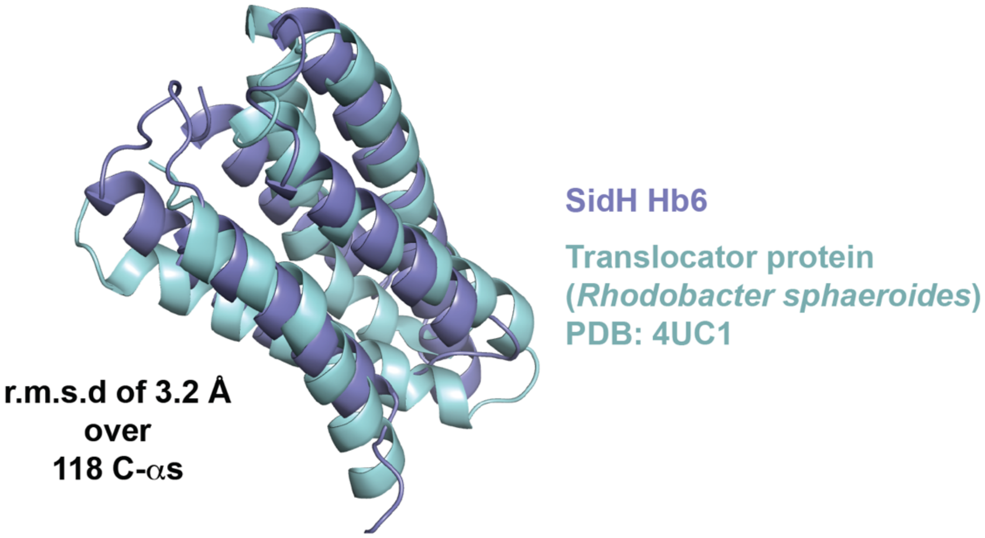
SidH Hb6
Translocator protein
(*Rhodobacter sphaeroides*)
PDB: 4UC1
r.m.s.d of 3.2 Å
over
118 C-αs

B)

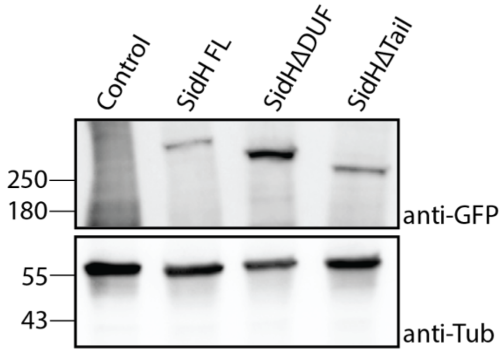
Control
SidH FL
SidHΔDUF
SidHΔTail
250
180
anti-GFP
55
43
anti-Tub

Figure S8- SidH interacts with the conserved T loop of tRNA

Alignment of different isotypes of *E. coli* tRNAs shows that nucleotides in T loop and acceptor arm are conserved. The conserved nucleotides are shown in white letters with red colored background. A cartoon representation of "L" shaped tRNA labelled with different parts is shown at the bottom.

Figure S8

E. coli tRNAs alignment

D loop — Anticodon loop — T loop — Acceptor arm

```
                    1        10         20         30                            40         50         60         70
chr.trna33-GluTTC   GTCCCCTTCGTCTAGAGGCCCAGGACACCGCCCT..................TTCACGGCGGTAACAGGGGTTCGAATCCCCTAGGGGACGCCA
chr.trna22-ArgCCT   GTCCTCTTAGTTA.AATGGATATAACGAGCCCCT..................CCTAAGGGCTAATTGCAGGTTCGATTCCTGCAGGGGACACCA
chr.trna62-GlyCCC   GCGGGCGT.AGTT.CAATGGTAGAACGAGAGCTT..................CCCAAGCTCTATACGAGGGTTCGATTCCCTTCGCCCGCTCCA
chr.trna45-GlyTCC   GCGGGCATCGTAT.AATGGCTATTACCTCAGCCT..................TCCAAGCTGATGATGCGGGTTCGATTCCCGCTGCCCGCTCCA
chr.trna80-SerTGA   GGAAGTGTGGCCGA.GCGGTTGAAGGCACCGGTCTTGAAAACCGGCGACCCG.....AAAGGGTTCCAGAGTTCGAATCTCTGCGCTTCCGCCA
chr.trna77-TyrGTA   GGTGGGGTTCCCGA.GCGGCCAAAGGGAGCAGACTGTAAATC........TGCCGTCATCGACTTCGAAGGTTCGAATCCTTCCCCCACCACCA
chr.trna73-SerCGA   GGAGAGATGCCGGA.GCGGCTGAACGGACCGGTCTCGAAAACCGGAGTAGG...GGCAACTCTACCGGGGGTTCAAATCCCCCTCTCTCCGCCA
chr.trna63-SerGCT   GGTGAGGTGGCCGA.GAGGCTGAAGGCGCTCCCCTGCTAAGGGAGTATGCGGTCAAAAGCTGCATCCGGGGTTCGAATCCCCGCCTCACCGCCA
chr.trna79-SerGGA   GGTGAGGTGTCCGA.GTGGCTGAAGGAGCACGCCTGGAAAGTGTGTATACGGCAA.....CGTATCGGGGGTTCGAATCCCCCCCTCACCGCCA
chr.trna53-LeuCAG   GCGAAGGTGGCGGA.ATTGGTAGACGCGCTAGCTTCAGGTGTTA......GTGTTCTTACGGACGTGGGGGTTCAAGTCCCCCCCCTCGCACCA
chr.trna60-LeuGAG   GCCGAGGTGGTGGA.ATTGGTAGACACGCTACCTTGAGGTGGTA......GTGCCCAATAGGGCTTACGGGTTCAAGTCCCGTCCTCGGTACCA
chr.trna76-LeuTAA   GCCCGGATGGTGGA.ATCGGTAGACACAAGGGATTTAAAATCCC......TCGGCGTTCGCGCTGTGCGGGTTCAAGTCCCGCTCCGGGTACCA
chr.trna51-LeuCAA   GCCGAAGTGGCGAA.ATCGGTAGACGCAGTTGATTCAAAATCAA......CCGTAGAA..ATACGTGCCGGTTCGAGTCCGGCCTTCGGCACCA
chr.trna83-LeuTAG   GCGGGAGTGGCGAA.ATTGGTAGACGCACCAGATTTAGGTTCTG......GCGCCGCA..AGGTGTGCGAGTTCAAGTCTCGCCTCCCGCACCA
chr.trna75-CysGCA   .GGCGCGTTAACAA.AGCGGTTATGTAGCGGATT.GCAAA.................TCCGTCTAGTCCGGTTCGACTCCGGAACGCGCCTCCA
chr.trna21-ProGGG   CGGCACGTAGCGCAGCCTGGTAGCGCACCGTCAT.GGGGTG................TCGGGGGTCGGAGGTTCAAATCCTCTCGTGCCGACCA
chr.trna56-ProCGG   CGGTGATTGGCGCAGCCTGGTAGCGCACTTCGTT.CGGGAC................GAAGGGGTCGGAGGTTCGAATCCTCTATCACCGACCA
chr.trna39-ProTGG   CGGCGAGTAGCGCAGCTTGGTAGCGCAACTGGTT.TGGGAC................CAGTGGGTCGGAGGTTCGAATCCTCTCTCGCCGACCA
chr.trna1-IleGAT    AGGCTTGTAGCTCAGGTGGTTAGAGCGCACCCCT.GATAAG................GGTGAGGTCGGTGGTTCAAGTCCACTCAGGCCTACCA
chr.trna8-ArgTCT    GCGCCCTTAGCTCAGTTGGATAGAGCAACGACCT.TCTAAG................TCGTGGGCCGCAGGTTCGAATCCTGCAGGGCGCGCCA
chr.trna64-ArgACG   GCATCCGTAGCTCAGCTGGATAGAGTACTCGGCT.ACGAAC................CGAGCGGTCGGAGGTTCGAATCCTCCCGGATGCACCA
chr.trna36-ArgCCG   GCGCCCGTAGCTCAGCTGGATAGAGCGCTGCCCT.CCGGAG................GCAGAGGTCTCAGGTTCGAATCCTGTCGGGCGCGCCA
chr.trna3-AspGTC    GGAGCGGTAGTTCAGTCGGTTAGAATACCTGCCT.GTCACG................CAGGGGGTCGCGGGTTCGAGTCCCGTCCGTTCCGCCA
chr.trna86-MetCAT   GGCTACGTAGCTCAGTTGGTTAGAGCACATCACT.CATAAT................GATGGGGTCACAGGTTCGAATCCCGTCGTAGCCACCA
chr.trna35-TrpCCA   AGGGGCGTAGTTCA.ATTGGTAGAGCACCGGTCT.CCAAAA................CCGGGTGTTGGGAGTTCGAGTCTCTCCGCCCCTGCCA
chr.trna50-GlyGCC   GCGGGAATAGCTCA.GTTGGTAGAGCACGACCTT.GCCAAG................GTCGGGGTCGCGAGTTCGAGTCTCGTTTCCCGCTCCA
chr.trna16-ValGAC   GCGTTCATAGCTCAGTTGGTTAGAGCACCACCTT.GACATG................GTGGGGGTCGTTGGTTCGAGTCCAATTGAACGCACCA
chr.trna30-PheGAA   GCCCGGATAGCTCA.GTCGGTAGAGCAGGGGATT.GAAAAT................CCCCGTGTCCTTGGTTCGATTCCGAGTCCGGGCACCA
chr.trna18-AsnGTT   TCCTCTGTAGTTCA.GTCGGTAGAACGGCGGACT.GTTAAT................CCGTATGTCACTGGTTCGAGTCCAGTCAGAGGAGCCA
chr.trna43-ThrTGT   GCCGACTTAGCTCA.GTAGGTAGAGCAACTGACT.TGTAAT................CAGTAGGTCACCAGTTCGATTCCGGTAGTCGGCACCA
chr.trna5-ThrCGT    GCCGATATAGCTCA.GTTGGTAGAGCAGCGCATT.CGTAAT................GCGAAGGTCGTAGGTTCGACTCCTATTATCGGCACCA
chr.trna59-ThrGGT   GCTGATATGGCTCA.GTTGGTAGAGCGCACCCTT.GGTAAG................GGTGAGGTCCCCAGTTCGACTCTGGGTATCAGCACCA
chr.trna87-GlnCTG   .TGGGGTATCGCCA.AGCGGTAAGGCACCGGATT.CTGATT................CCGGCATTCCGAGGTTCGAATCCTCGTACCCCAGCCA
chr.trna84-GlnTTG   .TGGGGTATCGCCA.AGCGGTAAGGCACCGGTTT.TTGATA................CCGGCATTCCCTGGTTCGAATCCAGGTACCCCAGCCA
chr.trna37-HisGTG   GTGGCTATAGCTCA.GTTGGTAGAGCCCTGGATT.GTGATT................CCAGTTGTCGTGGGTTCGAATCCCATTAGCCACCCCA
chr.trna11-LysTTT   GGGTCGTTAGCTCA.GTTGGTAGAGCAGTTGACT.TTTAAT................CAATTGGTCGCAGGTTCGAATCCTGCACGACCCACCA
chr.trna25-ValTAC   GGGTGATTAGCTCA.GCTGGGAGAGCACCTCCCT.TACAAG................GAGGGGGTCGGCGGTTCGATCCCGTCATCACCCACCA
chr.trna70-AlaGGC   GGGGCTATAGCTCA.GCTGGGAGAGCGCTTGCAT.GGCATG................CAAGAGGTCAGCGGTTCGATCCCGCTTAGCTCCACCA
chr.trna2-AlaTGC    GGGGCTATAGCTCA.GCTGGGAGAGCGCCTGCTT.TGCACG................CAGGAGGTCTGCGGTTCGATCCCGCATAGCTCCACCA
consensus>50        g.....t.g...a....g.taga........t....a...........................tc...gGTTCgA.tCc.........c.CCA
```

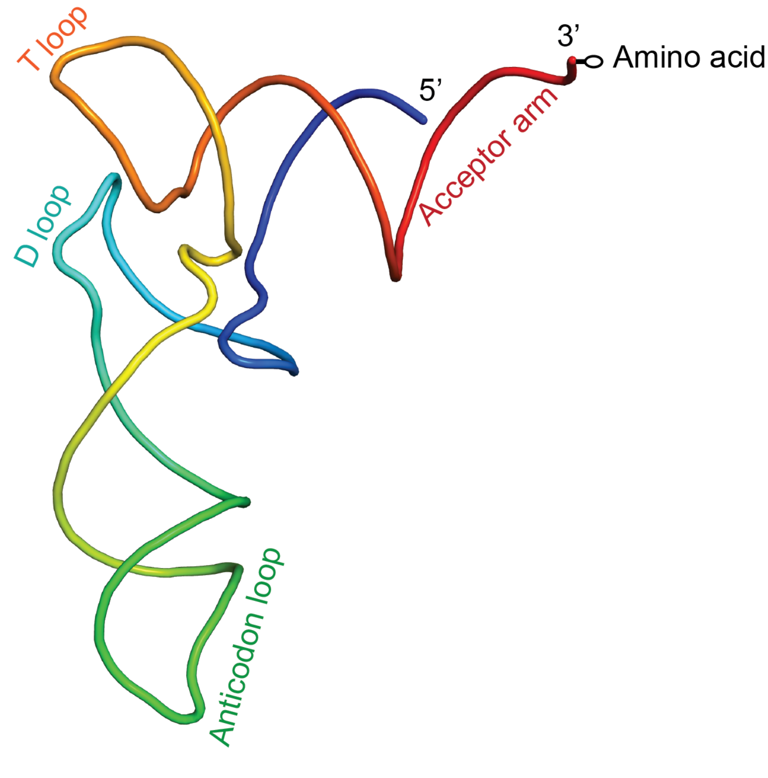

Figure S9- Graphical representation of the genomic context of the region containing the *sidH* gene in selected *L. pneumophila* strains

Protein-coding regions are represented by arrows (yellow arrows represent *sidH* genes). The gradient gray shading represents regions of nucleotide sequence identity (100%-64%) determined by BLASTn analysis. Figures are drawn to scale using Easyfig 2.2.2 (Sullivan et al., 2011)

Figure S9

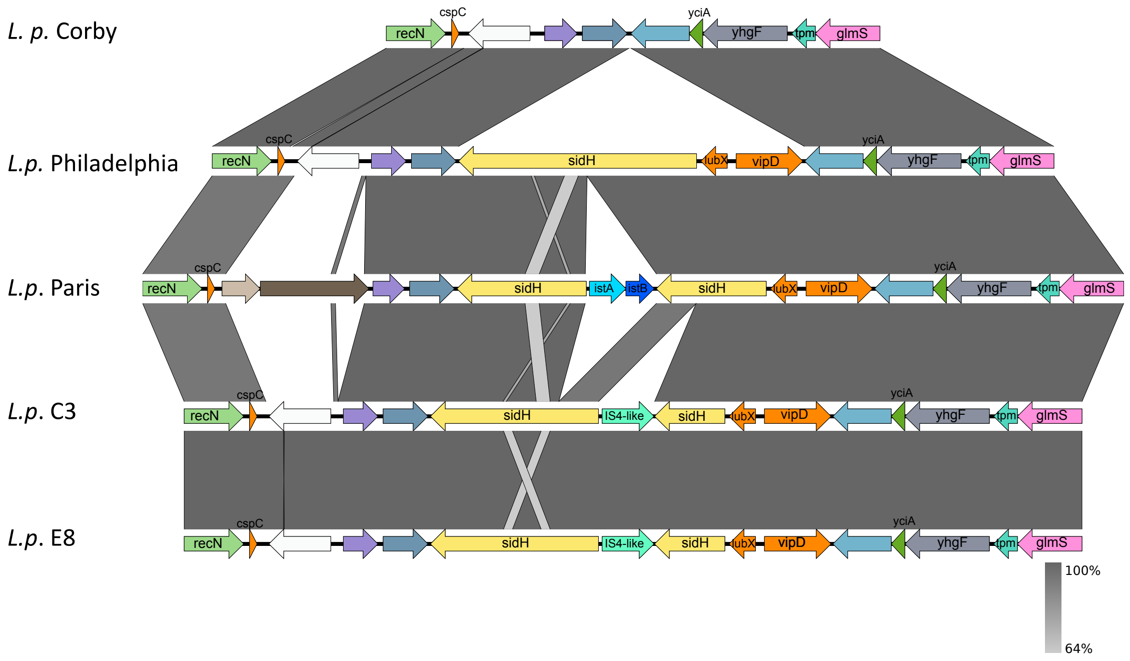
L. p. Corby
cspC
recN
yciA
yhgF
tpm
glmS
L.p. Philadelphia
cspC
recN
sidH
lubX
vipD
yciA
yhgF
tpm
glmS
L.p. Paris
cspC
recN
sidH
istA
istB
sidH
lubX
vipD
yciA
yhgF
tpm
glmS
L.p. C3
cspC
recN
sidH
IS4-like
sidH
lubX
vipD
yciA
yhgF
tpm
glmS
L.p. E8
cspC
recN
sidH
IS4-like
sidH
lubX
vipD
yciA
yhgF
tpm
glmS
100%
64%

Figure S10- Phylogenetic tree of 113 *L. pneumophila* strains and genomic region containing the *sidH* gene of strains representative of each cluster

Strains where *sidH* is present are shaded in green squares with black labels, strains where *sidH* is absent are shaded in red squares and have red labels. Strains where *sidH* is split due to transposase/s insertions (Paris, C3 and E8) are shaded in blue squares and have blue labels. The genomic context represented corresponds to the region between the genes *typA* and *glm*S and has been drawn using GeneSpy (PMID: 29912383). Genes are colored according to annotation files, *sidH* is always colored in burgundy. The phylogenetic tree was inferred using the fast core genome multialigner Parsnp (https://github.com/marbl/parsnp) and the option "force to include all genomes" was used to also include the *L. pneumophila* subspecies *pascullei* and *fraserii*. Numbers at nodes represent bootstrap support. The scale bar represents the estimated number of substitutions per site. Dashed lines highlighted in yellow represent branches artificially shortened for representation

Figure S10

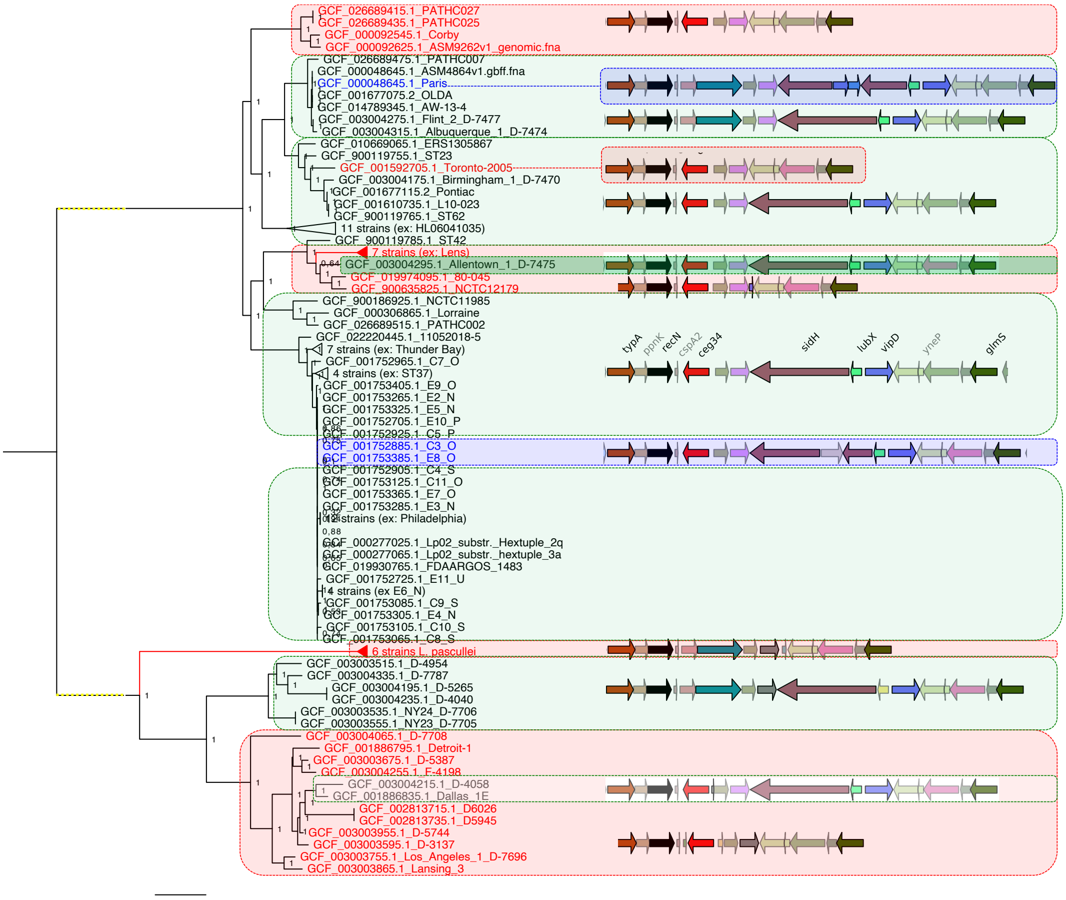

GCF_026689415.1_PATHC027
GCF_026689435.1_PATHC025
GCF_000092545.1_Corby
GCF_000092625.1_ASM9262v1_genomic.fna
GCF_026689475.1_PATHC007
GCF_000048645.1_ASM4864v1.gbff.fna
GCF_000048645.1_Paris
GCF_001677075.2_OLDA
GCF_014789345.1_AW-13-4
GCF_003004275.1_Flint_2_D-7477
GCF_003004315.1_Albuquerque_1_D-7474
GCF_010669065.1_ERS1305867
GCF_900119755.1_ST23
GCF_001592705.1_Toronto-2005
GCF_003004175.1_Birmingham_1_D-7470
GCF_001677115.2_Pontiac
GCF_001610735.1_L10-023
GCF_900119765.1_ST62
11 strains (ex: HL06041035)
GCF_900119785.1_ST42
7 strains (ex: Lens)
GCF_003004295.1_Allentown_1_D-7475
GCF_019974095.1_80-045
GCF_900635825.1_NCTC12179
GCF_900186925.1_NCTC11985
GCF_000306865.1_Lorraine
GCF_026689515.1_PATHC002
GCF_022220445.1_11052018-5
7 strains (ex: Thunder Bay)
GCF_001752965.1_C7_O
4 strains (ex: ST37)
GCF_001753405.1_E9_O
GCF_001753265.1_E2_N
GCF_001753325.1_E5_N
GCF_001752705.1_E10_P
GCF_001752925.1_C5_P
GCF_001752885.1_C3_O
GCF_001753385.1_E8_O
GCF_001752905.1_C4_S
GCF_001753125.1_C11_O
GCF_001753365.1_E7_O
GCF_001753285.1_E3_N
12 strains (ex: Philadelphia)
GCF_000277025.1_Lp02_substr._Hextuple_2q
GCF_000277065.1_Lp02_substr._hextuple_3a
GCF_019930765.1_FDAARGOS_1483
GCF_001752725.1_E11_U
4 strains (ex E6_N)
GCF_001753085.1_C9_S
GCF_001753305.1_E4_N
GCF_001753105.1_C10_S
GCF_001753065.1_C8_S
6 strains L. pascullei
GCF_003003515.1_D-4954
GCF_003004335.1_D-7787
GCF_003004195.1_D-5265
GCF_003004235.1_D-4040
GCF_003003535.1_NY24_D-7706
GCF_003003555.1_NY23_D-7705
GCF_003004065.1_D-7708
GCF_001886795.1_Detroit-1
GCF_003003675.1_D-5387
GCF_003004255.1_F-4198
GCF_003004215.1_D-4058
GCF_001886835.1_Dallas_1E
GCF_002813715.1_D6026
GCF_002813735.1_D5945
GCF_003003955.1_D-5744
GCF_003003595.1_D-3137
GCF_003003755.1_Los_Angeles_1_D-7696
GCF_003003865.1_Lansing_3
typA
ppnK
recN
cspA2
ceg34
sidH
lubX
vipD
yneP
glmS

Figure S11- Purification of N- and C-terminal of SidH^{Paris}

SEC (S200, 10/300 column) of A) SidH^{Paris} N-term, B) SidH^{Paris} N-term apo protein and C) SidH^{Paris} C-term. Labelled fractions were loaded onto 10% SDS-PAGE (right panel). The purification of SidH^{Paris} N-term and SidH^{Paris} C-term were performed atleast two independent times with similar results. Source data are provided as a Source Data file.

Figure S11

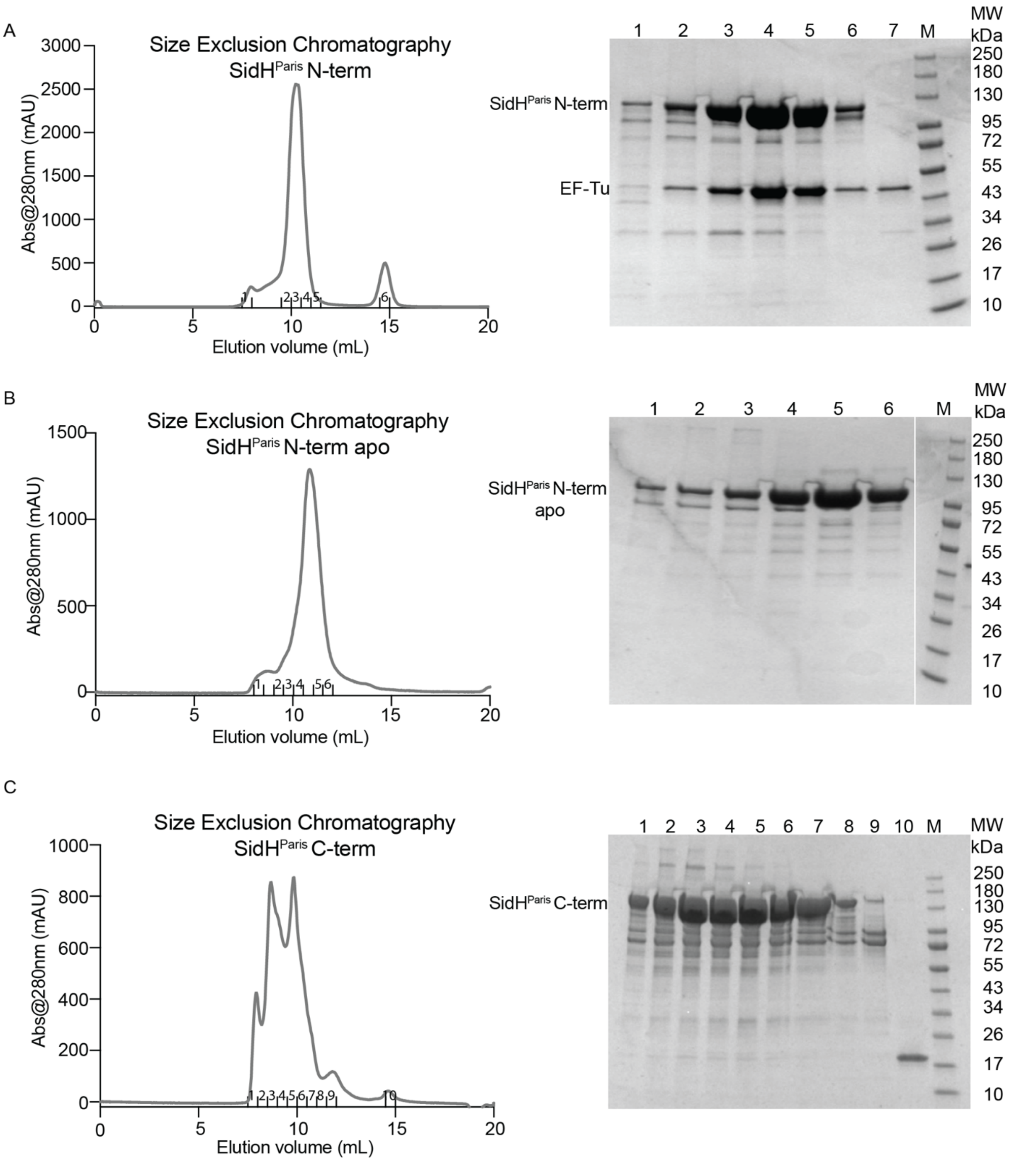
A
Size Exclusion Chromatography
SidH^Paris N-term
Abs@280nm (mAU)
Elution volume (mL)
0
500
1000
1500
2000
2500
3000
5
10
15
20
1 2 3 4 5 6 7 M
MW kDa
SidH^Paris N-term
EF-Tu
250
180
130
95
72
55
43
34
26
17
10
B
Size Exclusion Chromatography
SidH^Paris N-term apo
Abs@280nm (mAU)
Elution volume (mL)
1 2 3 4 5 6 M
MW kDa
SidH^Paris N-term apo
C
Size Exclusion Chromatography
SidH^Paris C-term
Abs@280nm (mAU)
Elution volume (mL)
200
400
600
800
1 2 3 4 5 6 7 8 9 10 M
MW kDa
SidH^Paris C-term

Figure S12-Toxic effect of SidH and mutant on the growth of HEK cells

A) Western blot data showing the expression of various SidH proteins in HEK cells corresponding to the toxicity assay shown in Figure 4C B) Expression of GFP-SidH WT/HM in HEK293T cells. Upper panel shows brightfield view and lower panel images were acquired after GFP excitation C) Western blot data showing the expression of various SidH proteins in HEK cells corresponding to the toxicity assay shown in Figure 5B D) Sample images of control HEK293T cells or cells transiently transfected with indicated GFP-tagged plasmids. Images were acquired using IncuCyte® S3 (10x) and show confluence (phase) or GFP signal at time point 72 h. E) Quantitative analysis of confluence shown in E comparing cell growth of control HEK293T cells or transiently transfected with indicated plasmids over a time period of 48 h. n=3. These experiments were performed at least three independent times with similar results. Source data are provided as a Source Data file for S12A, S12C, and S12E.

Figure S12

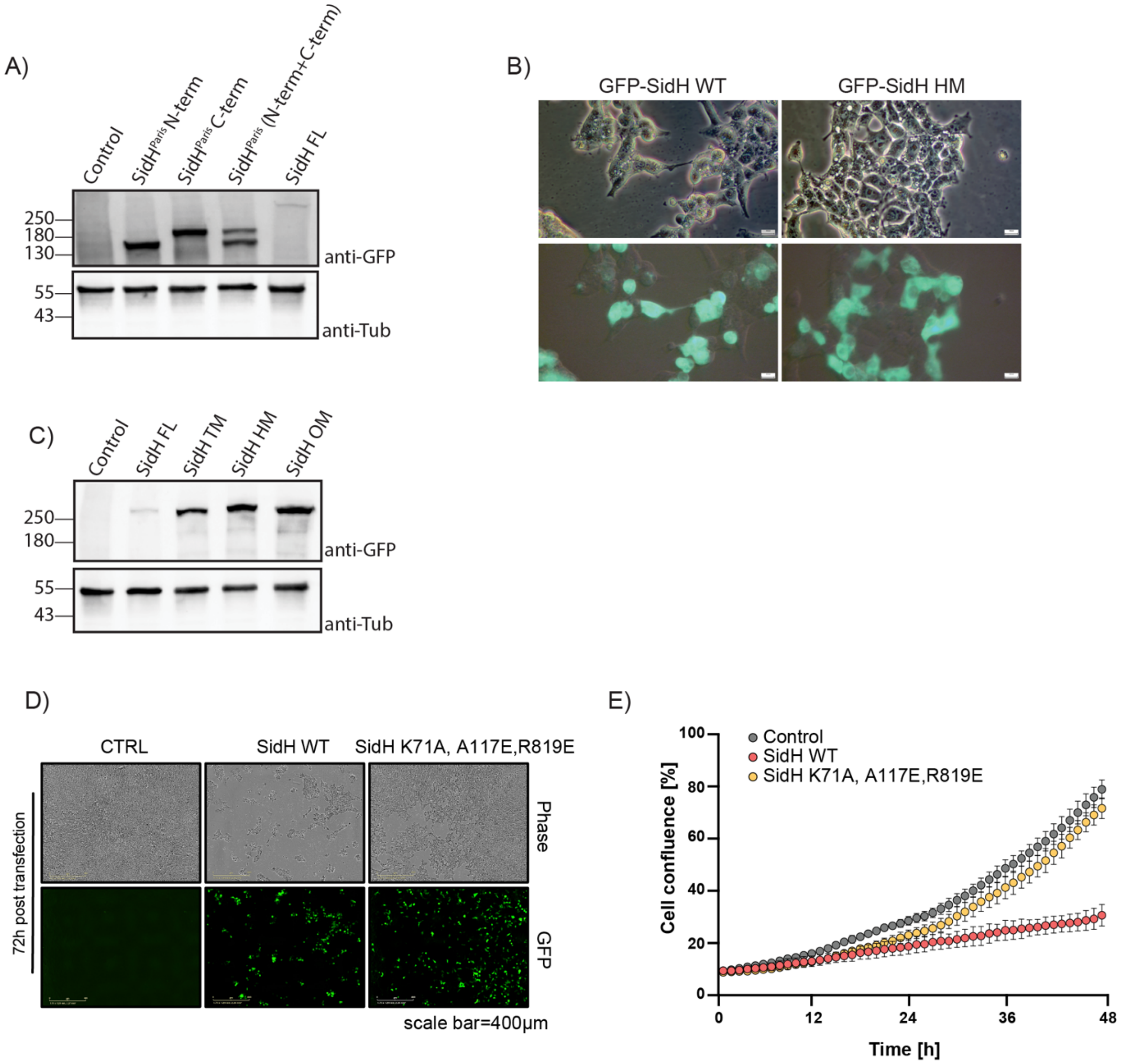
A)
Control
SidH^Paris N-term
SidH^Paris C-term
SidH^Paris (N-term+C-term)
SidH FL
250
180
130
anti-GFP
55
43
anti-Tub
B)
GFP-SidH WT
GFP-SidH HM
C)
Control
SidH FL
SidH TM
SidH HM
SidH OM
250
180
anti-GFP
55
43
anti-Tub
D)
CTRL
SidH WT
SidH K71A, A117E,R819E
72h post transfection
Phase
GFP
scale bar=400µm
E)
Control
SidH WT
SidH K71A, A117E,R819E
Cell confluence [%]
Time [h]
0
12
24
36
48
20
40
60
80
100

Figure S13- Flow chart of SidH-LubX complex preparation

A) Affinity chromatography fractions on SDS-PAGE gel: From left to right: Marker (M), flow through (FT), wash (W), 10 mM Imidazole elution (E10), 100 mM Imidazole elution (E100), 200 mM Imidazole elution (E200), 300 mM Imidazole elution (E300), talon beads (B). The 100 mM imidazole elution fraction is boxed. B) SEC of 100 mM Imidazole elution fraction shows the elution profile. All the peak fractions labelled with blue bar were loaded onto gel (right panel). One fraction boxed in orange rectangle was used for gradient fixation. The highlighted SEC fraction was cross-linked via GraFix method. C) Silver stained SDS-PAGE gel of all the fractions. Highlighted fractions were pooled and used to make grids. The purification was performed atleast two independent times with similar results. Source data are provided as a Source Data file.

Figure S13

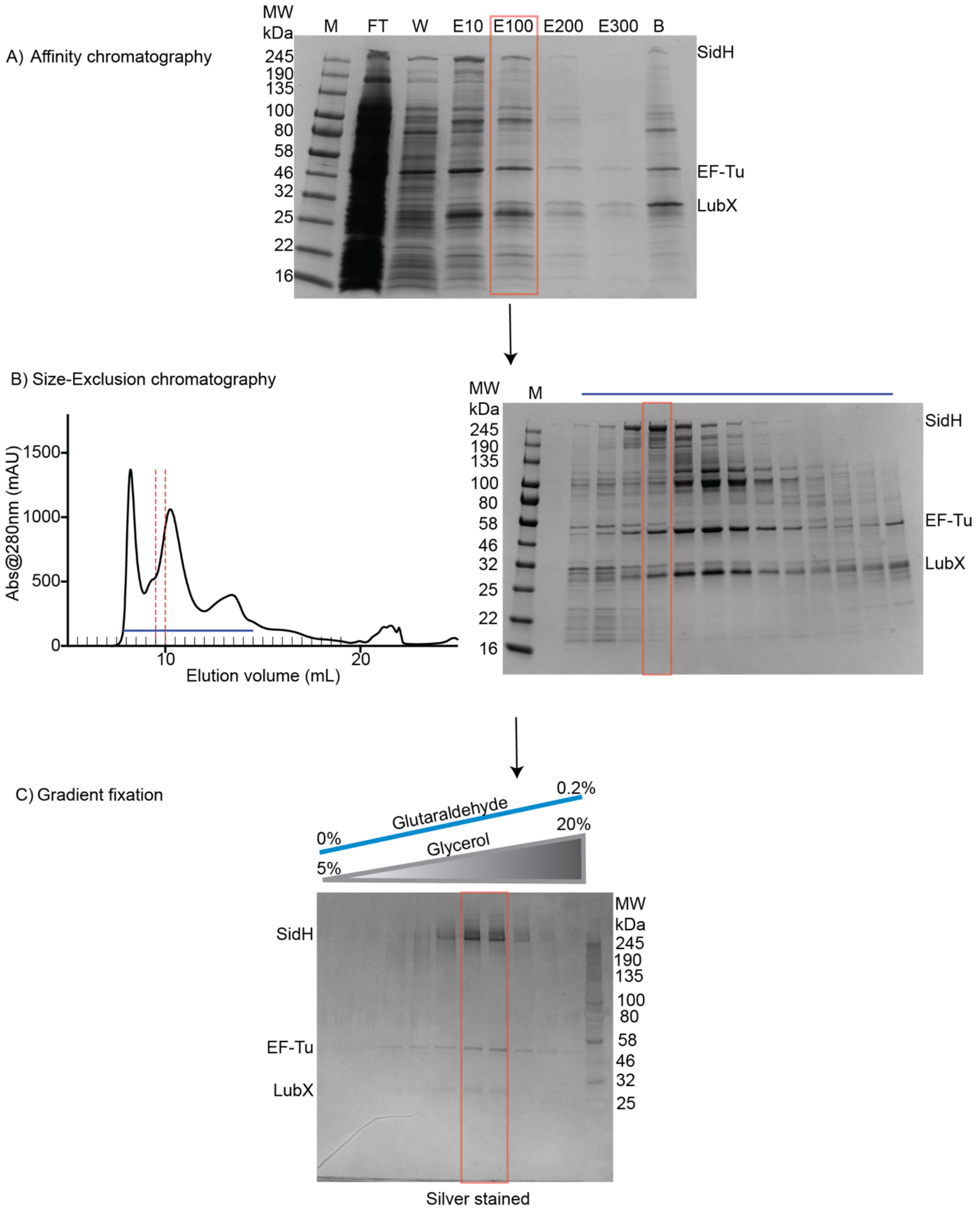
A) Affinity chromatography
MW kDa
M FT W E10 E100 E200 E300 B
245
190
135
100
80
58
46
32
25
22
16
SidH
EF-Tu
LubX
B) Size-Exclusion chromatography
Abs@280nm (mAU)
1500
1000
500
0
10
20
Elution volume (mL)
MW kDa
M
245
190
135
100
80
58
46
32
25
22
16
SidH
EF-Tu
LubX
C) Gradient fixation
0%
Glutaraldehyde
0.2%
5%
Glycerol
20%
SidH
EF-Tu
LubX
MW kDa
245
190
135
100
80
58
46
32
25
Silver stained

Figure S14- cryo-EM processing pipeline of SidH-LubX complex

Particles were picked using WARP and processed using RELION 3.0, pipeline shows models upto post-processing step. Bottom left, Gold Standard FSC curves showing a final resolution of 3.06 Å.

Figure S14

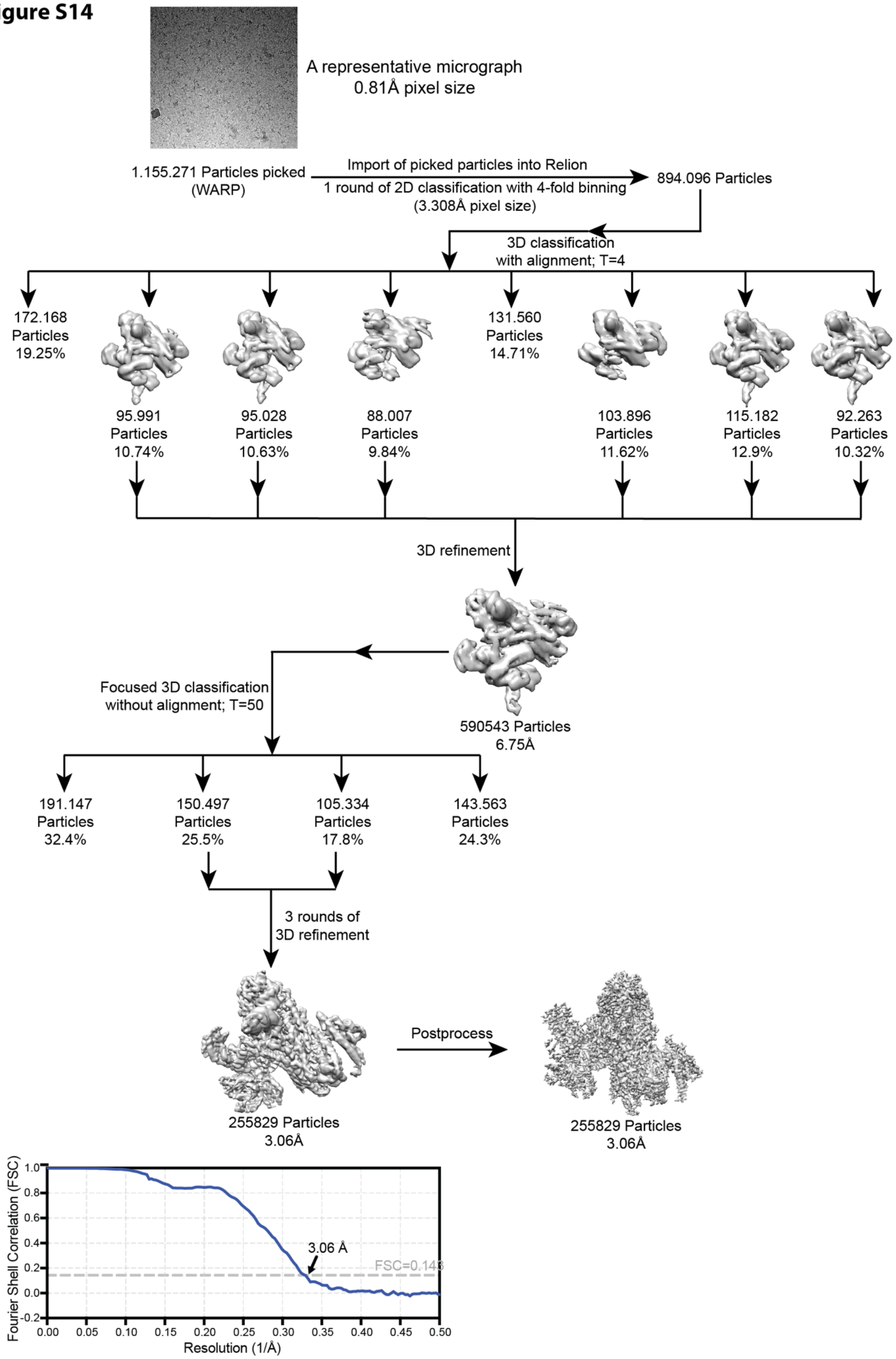

A representative micrograph
0.81Å pixel size
1.155.271 Particles picked
(WARP)
Import of picked particles into Relion
1 round of 2D classification with 4-fold binning
(3.308Å pixel size)
894.096 Particles
3D classification
with alignment; T=4
172.168
Particles
19.25%
95.991
Particles
10.74%
95.028
Particles
10.63%
88.007
Particles
9.84%
131.560
Particles
14.71%
103.896
Particles
11.62%
115.182
Particles
12.9%
92.263
Particles
10.32%
3D refinement
590543 Particles
6.75Å
Focused 3D classification
without alignment; T=50
191.147
Particles
32.4%
150.497
Particles
25.5%
105.334
Particles
17.8%
143.563
Particles
24.3%
3 rounds of
3D refinement
255829 Particles
3.06Å
Postprocess
255829 Particles
3.06Å
Fourier Shell Correlation (FSC)
1.0
0.8
0.6
0.4
0.2
0.0
-0.2
3.06 Å
FSC=0.143
0.00
0.05
0.10
0.15
0.20
0.25
0.30
0.35
0.40
0.45
0.50
Resolution (1/Å)

Figure S15- SidH ubiquitination via LubX

A) C-terminal helix of LubX is resolved in the presence of SidH- Overlap of crystallographic LubX-UBE2D2 (PDB: 4WZ3) structure with cryo-EM structure of LubX-SidH, detailed view is shown on right panel inset. B) *In vitro* ubiquitination reaction showing that LubX is able to ubiquitinated WT SidH as well as SidH HM which is deficient in binding to tRNA and EF-Tu. Respective reaction mixtures were analyzed by coomassie staining (Top), western blotting using Ubiquitin antibody (Middle), and western blotting using GST antibody to detect LubX. The ubiquitination experiment was performed atleast two independent times with similar results. Source data are provided as a Source Data file.

Figure S15

A)

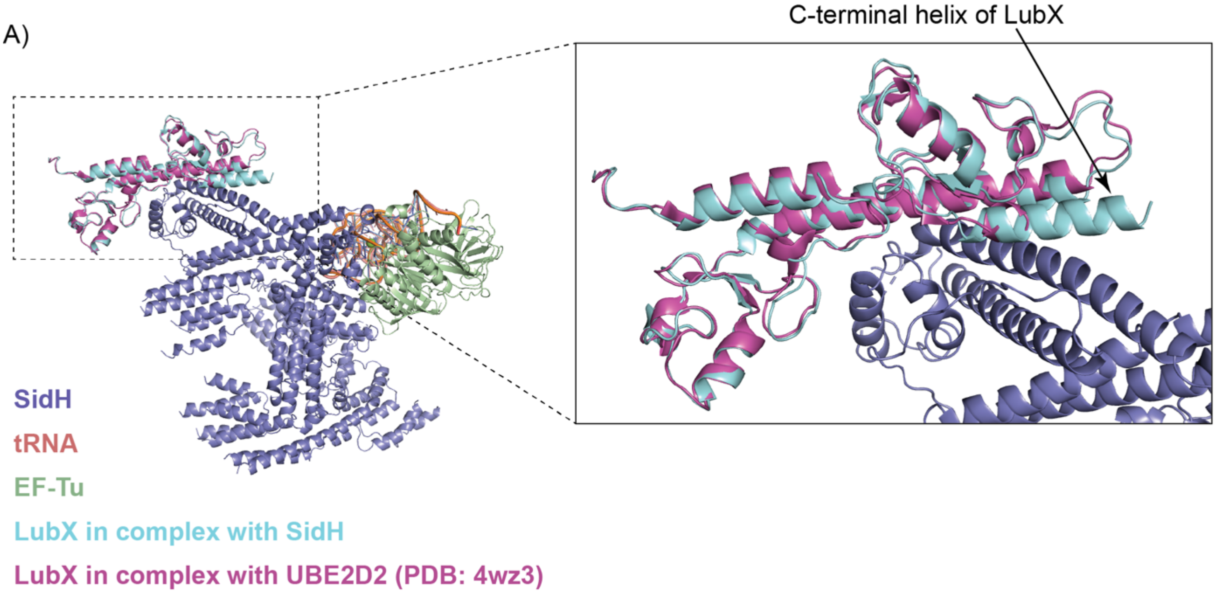

B)

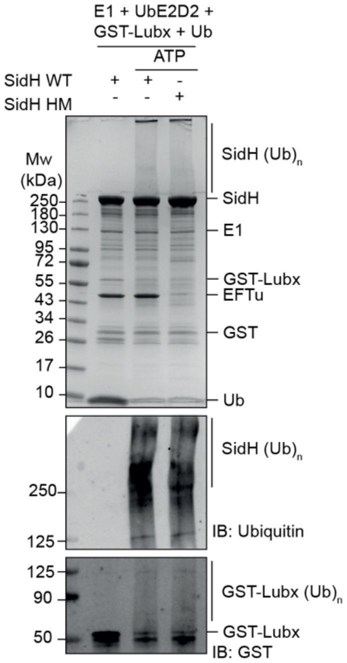

Figure S16- Multiple sequence alignment of SdhA family homologues

The sequence alignment was performed using Clustal Omega for N-terminal 700 amino acids of the SdhA family members. The residues responsible for binding to tRNA, EF-Tu and LubX are boxed with yellow, green and red rectangles respectively.

Figure S16

```
                10        20        30        40        50        60
SidH    1 MKRTIETYIIYLKEDLKIADTC----------KTIKDGLLKSITDKTHFSEELATYFERDN-PNAPFKVN  59
SdhB    1 -------MLLLSIEVINMPDHNSSDIPLSQQIDEVRGLL-LKAL-KAQLDDLIAGKL-TPQPSGVPFVHF  60
SdhA    1 ----------MISEKIKLLESFK-QNQFSKLIEKINSKI-LATLKSGHFNPRLAPYLEKMNKDGVPFTNF  58

                80        90        100       110       120       130
SidH   60 TTDPTQVAVLKKLLNALENAEKSFRAIENIDISRDRYTAMIAKDAVMVSYKAVHEIYAALQLINHSNSDI 129
SdhB   61 EKDPPQIATLKKVINCLYHAEEAFKSWESIDTSTLLGKAKAAPKL----IRALTQIYKSLGLLDEATPEI 126
SdhA   59 DLDPENISQIKKLINALYHARLAFLDLENIDIGNIKRTI---PDLKLLYSNTIHQAYQAGYLITNLDVDL 125

                150       160       170       180       190       200
SidH  130 QDIVGPHIQKLLPQMALASKALG--NFAPEHPEESA--------------GAVLAGV--VNMLPTEKPTE 181
SdhB  127 RSVIADNYHLLEPLFTQAYTIVQESGWLSEFMEMDV----TDKASKVIFQGMDLLG----------PDID 182
SdhA  126 QEMFKEEIDLILPVIGKLQ------AFAGEHQECTKRLAESWKDFPISYKAGEVTGIALEQMQPRTGD-- 187

                220       230       240       250       260       270
SidH  182 SESLGKLSNLIFELPHYFEELQKLIATGASGIATKSITSA----EDYQSAMIKKANETKYYFEQLSSKSG 247
SdhB  183 KWHDTH------PLVDAFAKISKLVETISKLQEKDMPGKEKEQAVELVRAL----------LDDLDNNPF 236
SdhA  188 -WDYQFLTQFSAVLPGYIEKLTQYIQQYSSQIKEKEPTLNNEKLEELQNASLKLLND----LEHLKGNDF 252

                290       300       310       320       330       340
SidH  248 --LLAIPSYL--SIVKRLIAHSTDLVNAGAPLTKQAYLDAVAKLEDIKHNILPQLISELEMVEESMGLKP 313
SdhB  237 ISKLSISDFEDSKAIKDLLEWFKNIQDDGFDFSKKSI----KQYVSWANHYLPTLITFADQLERQNYLKP 302
SdhA  253 FISLKVLKYI--HIIRNIITLATSSVEQMGHFSDSSQDVIRDNLAQLKYVVLPTLFGLVDKIEDNAMLKP 320

                360       370       380       390       400       410
SidH  314 GLLTDPALEQMNKYYTQLAEQVDNIAKAAGVLDTVSDYSDSIGGKIVRFLAGDSKKLDVGPKLTPAPDLG 383
SdhB  303 GSLSTNLCSQIEHLGKQVNGILSEP---------------------------------------NFG 330
SdhA  321 GTLSIPLMEKIKPLYQLLIYYASKP--------------------VNFKE-------KGEELL------ 356

                430       440       450       460       470       480
SidH  384 VLMDDVFIQKRRSNQESRLNEAR----LSSE-DKSVLAAANRFFDKIGSYNSIHKAWSKWSLANISQSEK 448
SdhB  331 ITERVVSIESLKGKREQQIHNAQANSVRAIMATEKQQAATADFYRILEAYKGMN------LSQVTEPDR 393
SdhA  357 SIEDSRFLALRLERTYQRIDEAN----KALFKIQKAQEALDNFYKILD-----TPPYNKQPIHQLPKEIK 417

                500       510       520       530       540       550
SidH  449 DALIKEYKQFQPHFAALYPDIDKLVVDAL--TQPTGSNIVSRLYSSDYKQLWS-------------SDH 502
SdhB  394 ILLRQLYPQIQTALAHASLDLENLLTSVLNTTGPEKT-------PAKPKSWWELAKGGLGYVASYVVNYN 456
SdhA  418 NQLITYYKIISPYMMDVDPDLNILLIDSF--QGPESW-------SSYLKKPWRWLRGTLP-------ADH 471

                570       580       590       600       610       620
SidH  503 FKQVLSCKDSVLSSIQQSLAQSEFKAK----LIEKTMSH---SEETAYSMNNK--------TTNLTTRVQ 557
SdhB  457 VDKILGTRESVDRFLSSQIASEQFKILIAEKAREKLRTGSDKLEETALQYKAEVRVNAIKSTLRKQPLQR 526
SdhA  472 ASFVLAQKEALQTLISKKKATQQFHIELNRDLIESVHKQ---TNLVLFPYSEKTNVFLLDETKALNPANG 538

                640       650       660       670       680       690
SidH  558 PFEPLKFTLEDDKPVEYYHKRVIAASNQILEL----------------ERAQKGVAEFFNYI-------- 603
SdhB  527 EAPPSTFVAVKPSKLV-N-LRGTLTAIQEMQLSTTVNSTRTNVDTLIHRHLPEKYQACFSTLPYQVNEKD 594
SdhA  539 ATDKLKFRPEKGHNILVNPED--LTSEQALDLYQWYRNKREKF-----RVASKAYNEFITLL-------- 593

                710       720       730       740       750       760
SidH  604 ---QKKYP----------HENPSFDSLDESDKEFLRKAYKTFQPQLLALKHDDINTR-------LVSSLT 653
SdhB  595 PELVKQIKTIENNLFQLEKALKDFEKIDLSYGIMIRL------HYFIAIASAASK------LKSSVKELS 652
SdhA  594 ---KKQTKTIA----ETEGRILHLNNLDKETKTRCRNLYNIFQPYFIDGIPPELRASAISFDKFLVHSFS 656

                780       790       800       810       820
SidH  654 SSK--PTDPPLRLTDLVSLKSGIND--YLNEKISDLNQDKTTLLDKEEEAR----            700
SdhB  653 PEGQKALAPLL--QQIMTLGSSFSDTNYTTNDLSAL-----QQLKTEGQARPGSP            700
SdhA  657 NESSTYDAPAVDLFE--KLDEHFQI--YFTEIDLQWGKKSNQYLKWAQ-------            700
```

tRNA-binding   EF-Tu-binding   LubX-binding

Figure S17- Potential ubiquitination sites on SidH and expression of SidH/LubX constructs in HEK cells

A) A cartoon representation of SidH divided into various helical bundles. We highlighted the identified lysines that are targeted for ubiquitination by LubX. B) Expression level of different proteins in HEK293T cells: Western blot analysis of expression levels of $SidH^{Paris}$ C-term (using GFP- antibody), $SidH^{Paris}$ N-term (using HA- antibody), LubX (using mCherry-antibody) in HEK293T cells. The loading control tubulin was detected using Anti-Tub antibody. On the right, similar analysis was carried out for the expression of SidH Philadelphia ($SidH^{Phila}$) protein. This experiment was performed at least three independent times with similar results. Source data are provided as a Source Data file.

Figure S17

A)

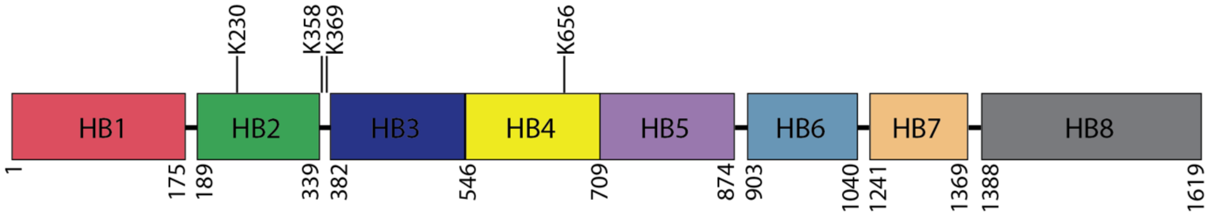

B)

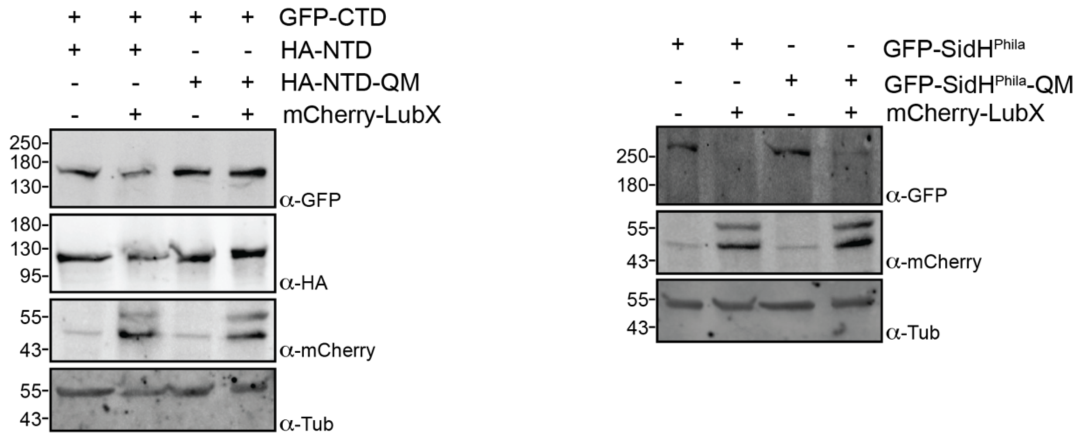

Figure S18- Infection of *Acanthamoeba castellani* with *L. pneumophila* WT strain Paris or the Δ*lpp2886* mutant

A) Growth curves (black- WT; green- Δ*lpp2886*) show comparable growth over different time points post infection. n=3 B) A protein expression control for the experiment shown in Figure 7B. Western blot experiment showing the expression of various SidH proteins in *L. pneumophila* Paris strain. This experiment was performed at least two independent times with similar results. Source data are provided as a Source Data file.

Figure S18

A)

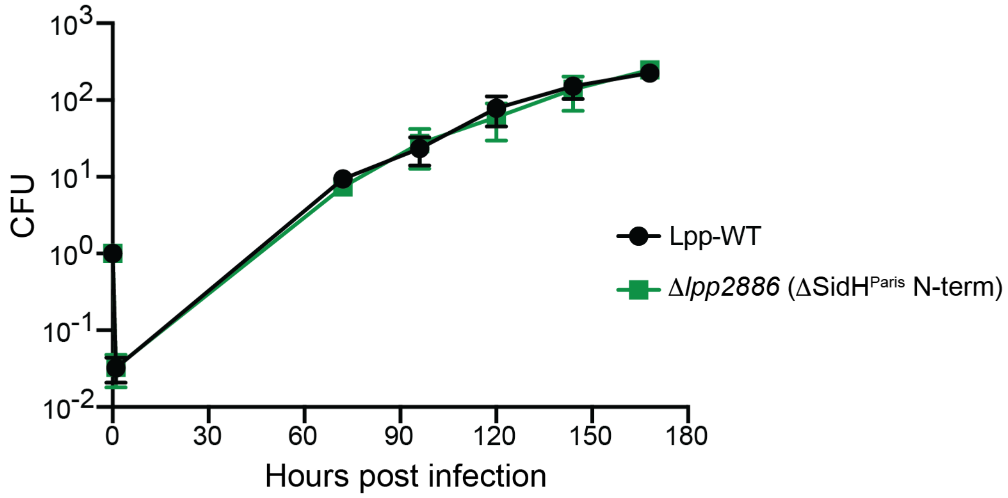

B)

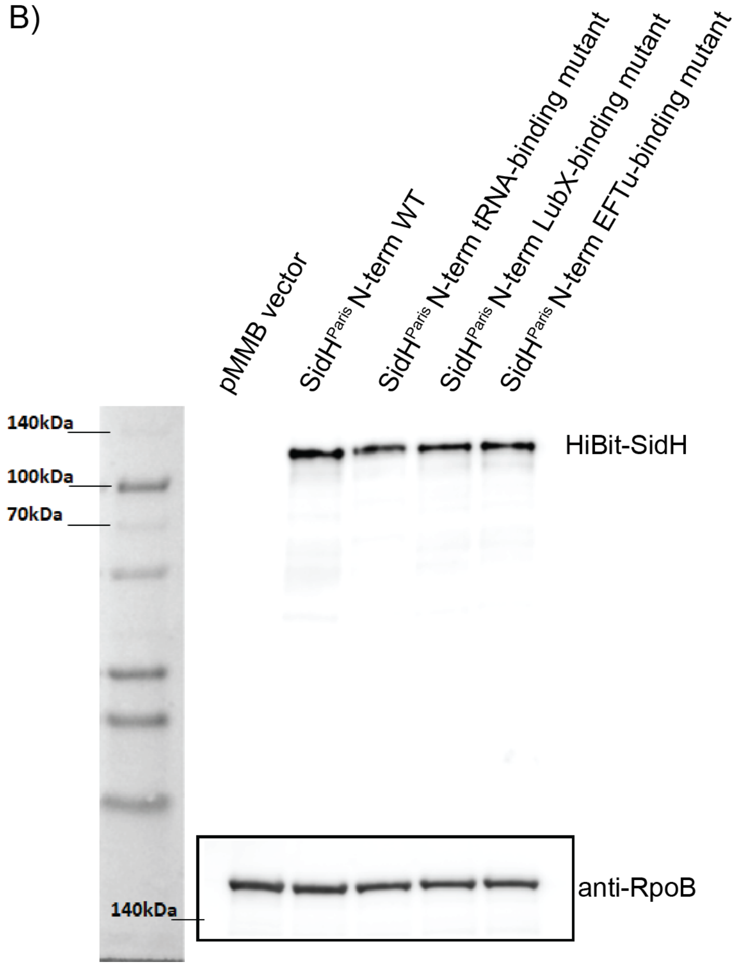

Figure S19: Sequence alignment of E.coli EF-Tu with the human Elongation factor Tu, mitochondrial (TUFM) with the conserved residues highlighted as indicated.

Figure S19

Alignment between EF-Tu_E. coli and TUFM_Human

```
EFTu_E.coli       1 --------------------------------------------------MS      2
                                                                    .:
TUFM_Human        1 MTTMAAATLLRATPHFSGLAAGRTFLLQGLLRLLKAPALPLLCRGLAVEA     50

EFTu_E.coli       3 KEKFERTKPHVNVGTIGHVDHGKTTLTAAITTVLAKTYGGAARAFDQIDN     52
                    |:.:.|.||||||||||||||||||||||||.:||:..|...:.:::|||
TUFM_Human       51 KKTYVRDKPHVNVGTIGHVDHGKTTLTAAITKILAEGGGAKFKKYEEIDN    100

EFTu_E.coli      53 APEEKARGITINTSHVEYDTPTRHYAHVDCPGHADYVKNMITGAAQMDGA    102
                    ||||:|||||||.:||||.|..|||||.|||||||||||||||.|.:||.
TUFM_Human      101 APEERARGITINAAHVEYSTAARHYAHTDCPGHADYVKNMITGTAPLDGC    150

EFTu_E.coli     103 ILVVAATDGPMPQTREHILLGRQVGVPYIIVFLNKCDMVDDEELLELVEM    152
                    ||||||.||||||||||:||.||:||.:::|::||.|.|.|.|::||||:
TUFM_Human      151 ILVVAANDGPMPQTREHLLLARQIGVEHVVVYVNKADAVQDSEMVELVEL    200

EFTu_E.coli     153 EVRELLSQYDFPGDDTPIVRGSALKALEG-DAEWEAK-ILELAGFLDSYI    200
                    |:||||:::.:.|::||::.||||.|||| |.|...| :.:|...:|:||
TUFM_Human      201 EIRELLTEFGYKGEETPVIVGSALCALEGRDPELGLKSVQKLLDAVDTYI    250

EFTu_E.coli     201 PEPERAIDKPFLLPIEDVFSISGRGTVVTGRVERGIIKVGEEVEIVGIKE    250
                    |.|.|.::||||||:|.|:|:.||||||||.:||||:|.|:|.|::|..:
TUFM_Human      251 PVPARDLEKPFLLPVEAVYSVPGRGTVVTGTLERGILKKGDECELLGHSK    300

EFTu_E.coli     251 TQKSTCTGVEMFRKLLDEGRAGENVGVLLRGIKREEIERGQVLAKPGTIK    300
                    ..::..||:|||.|.|:...||:|:|.|:||:|||::.||.|:.|||:||
TUFM_Human      301 NIRTVVTGIEMFHKSLERAEAGDNLGALVRGLKREDLRRGLVMVKPGSIK    350

EFTu_E.coli     301 PHTKFESEVYILSKDEGGRHTPFFKGYRPQFYFRTTDVTGTIELPEGVEM    350
                    ||.|.|::||||||:|||||.||...:.|..:..|.|:...|.||...|:
TUFM_Human      351 PHQKVEAQVYILSKEEGGRHKPFVSHFMPVMFSLTWDMACRIILPPEKEL    400

EFTu_E.coli     351 VMPGDNIKMVVTLIHPIAMDDGLRFAIREGGRTVGAGVVAKVLS------    394
                    .|||:::|..:.|..|:.::.|.||.:|:|.||:|.|:|...|:
TUFM_Human      401 AMPGEDLKFNLILRQPMILEKGQRFTLRDGNRTIGTGLVTNTLAMTEEEK    450

EFTu_E.coli     395 -----    394

TUFM_Human      451 NIKWG    455
```

Conserved Residues

Hydrophobic/charged residue replaced with another hydrophobic/charged residue

Non-conserved Residues

Figure S20: Sequence alignment of E.coli EF-Tu with the human Elongation factor 1-alpha 1 (EEF1A1) with the conserved residues highlighted as indicated.

Figure S20

Alignment between EF-Tu_E. coli and EEF1A1_Human

```
EFTu_E.coli       1 MSKEKFERTKPHVNVGTIGHVDHGKTTLTAAITTVLAKTYGGAARAFDQ-     49
                    |.||     |.|:|:..|||||.||:|.|..:   :.|..|...|..::
EEF1A1_Human      1 MGKE-----KTHINIVVIGHVDSGKSTTTGHL---IYKCGGIDKRTIEKF     42

EFTu_E.coli      50 -----------------IDNAPEEKARGITINTSHVEYDTPTRHYAHVDC     82
                                     :|....|:.|||||:.|..:::|...:...:|.
EEF1A1_Human     43 EKEAAEMGKGSFKYAWVLDKLKAERERGITIDISLWKFETSKYYVTIIDA     92

EFTu_E.coli      83 PGHADYVKNMITGAAQMDGAILVVAATDGPMP-------QTREHILLGRQ    125
                    |||.|::||||||.:|.|.|:|:|||..|...       |||||.||...
EEF1A1_Human     93 PGHRDFIKNMITGTSQADCAVLIVAAGVGEFEAGISKNGQTREHALLAYT    142

EFTu_E.coli     126 VGVPYIIVFLNKCDMVDD-------EELLELVEMEVRELLSQYDF-----    163
                    :||..:||.:||.|..:.       ||:::.|...::::....|.
EEF1A1_Human    143 LGVKQLIVGVNKMDSTEPPYSQKRYEEIVKEVSTYIKKIGYNPDTVAFVP    192

EFTu_E.coli     164 -----------PGDDTPIVRGSALKALEGDAEWEAKILELAGFLDSYIPE    202
                               |..:.|..:|..:...:|:|. ...:||.   ||..:|
EEF1A1_Human    193 ISGWNGDNMLEPSANMPWFKGWKVTRKDGNAS-GTTLLEA---LDCILP-    237

EFTu_E.coli     203 PERAIDKPFLLPIEDVFSISGRGTVVTGRVERGIIKVGEEVEIVGIKETQ    252
                    |.|..|||..||::||:.|.|.|||..||||.|::|.|..|....:..|
EEF1A1_Human    238 PTRPTDKPLRLPLQDVYKIGGIGTVPVGRVETGVLKPGMVVTFAPVNVT-    286

EFTu_E.coli     253 KSTCTGVEMFRKLLDEGRAGENVGVLLRGIKREEIERGQVLAKPGTIKP-    301
                     :....|||..:.|.|...|:|||..::.:..:::.||.|........|
EEF1A1_Human    287 -TEVKSVEMHHEALSEALPGDNVGFNVKNVSVKDVRRGNVAGDSKNDPPM    335

EFTu_E.coli     302 -HTKFESEVYILSKDEGGRHT-PFFKGYRPQFYFRTTDVTGTI-------    342
                     ...|.::|.||:      |. ....||.|.....|..:....
EEF1A1_Human    336 EAAGFTAQVIILN------HPGQISAGYAPVLDCHTAHIACKFAELKEKI    379

EFTu_E.coli     343 ------ELPEGVEMVMPGDNIKMVVTLI--HPIAMDD-------GLRFAI    377
                          :|.:|.:.:..||  ..:|.::  .|:.::.       | |||:
EEF1A1_Human    380 DRRSGKKLEDGPKFLKSGD--AAIVDMVPGKPMCVESFSDYPPLG-RFAV    426

EFTu_E.coli     378 REGGRTVGAGVVAKVLS-------------------     394
                    |:..:||..||:..|..
EEF1A1_Human    427 RDMRQTVAVGVIKAVDKKAAGAGKVTKSAQKAQKAK     462
```

Conserved Residues (green box)

Hydrophobic residue replaced with another hydrophobic residue (yellow box)

Non-conserved Residues (red box)

Supplementary table 1: cryo-EM data collection, refinement and validation statistics

**Data collection and processing**		
	**SidH**	**SidH-LubX**
Magnification	165k	165k
Voltage (kV)	300	300
Electron exposure (e-/Å^2)	41.93	43.36
Defocus range (μm)	-0.8 to -2.0	-0.8 to -2.2
Pixel size (Å)	0.81	0.827
Symmetry imposed	C1	C1
Initial particle images (no.)	1.370.015	1.155.271
Final particle images (no.)	367.741	255.829
Map resolution (Å)	2.72	3.06
FSC threshold	0.143	0.143
Map resolution range (Å)	2.5-6.0	2.7-6.0
**Refinement**		
	**SidH**	**SidH-LubX**
Refinement resolution limit (Å)	2.72	3.06
Model resolution range (Å)	2.5-6.0	2.7-6.0
Map sharpening B factor (Å^2)	-79.94	-99.57
Initial models used (PDB)	1TTT and *ab initio*	SidH and 4WZ3
**Model composition**		
Non-hydrogen atoms	13691	15288
Protein residues	1583	1778
Nucleotide	76	76
Ligands	3	4
**B factors (Å^2)**		
Protein	66.75	57.56
Nucleotide	17.56	21.97
Ligand	36.21	25.84
**R.m.s. deviations**		
Bond lengths (Å)	0.01 (54)	0.007 (30)
Bond angles (degrees)	1.208 (107)	1.093 (16)
**Validation**		
	**SidH**	**SidH-LubX**
MolProbity score	1.74	1.72
Clashscore	9.88	10.49
Rotamer Outliers (%)	0.00	0.07
**Ramachandran plot (%)**		
Favored	96.52	96.96
Allowed	3.35	2.98
Disallowed	0.13	0.06

Supplementary table 2: Primers used for site-directed mutagenesis

Mutation Site	Forward Primer	Reverse Primer
SidHΔDUF	agtggaagtggaagtggaagtggatcactgaaagatacttcttatttacaattaattgcagaaagc	tccacttccacttccacttccacttccttttgacaaaccagattctacaaccg
SidHΔTail	cttgtattttttctggctaaacttccagctcctgtttctttagcagat	atctgctaaagaaacaggagctggaagtttagccagaaaaaatacaag
SidH_K57E	gtaggatcagtcgtgttaacctcaaaaggggcattggggttat	ataaccccaatgccccttttgaggttaacacgactgatcctac
SidH_K71E	attttccagagcattaagcaactctttcagaacagcgacttgggtag	ctacccaagtcgctgttctgaaagagttgcttaatgctctggaaaat
SidH_K110E	cataaatttcatgcaccgcctcataactgaccatgactgcatctttagc	gctaaagatgcagtcatggtcagttatgaggcggtgcatgaaatttatg
SidH_A117E	taaagcggtgcatgaaatttatgaggctcttcaactaatcaatc	gattgattagttgaagagcctcataaatttcatgcaccgcttta
SidH_K504E	caggataatacctgctcaaaatgatcagaactccataattgcttataa	ttataagcaattatggagttctgatcattttgagcaggtattatcctg
SidH_R819E	ccccaataacatttatcaccgtacggagtttgtcatgaccacatttaatgctc	gagcattaaatgtggtcatgacaaactccgtacggtgataaatgttattgggg
SidH_W438A	ttcacaaagcatggtccaaggcgagtttggcaaacattagtc	gactaatgtttgccaaactcgccttggaccatgctttgtgaa
SidH_V486E	cctaccggaagcaatatcgagtccagactatacagttccg	cggaactgtatagtctggactcgatattgcttccggtagg
SidH_S499E	tatacagttccgattataagcaattatgggagtctgatcattttaagcaggtattatcctg	caggataatacctgcttaaaatgatcagactcccataattgcttataatcggaactgtata
SidH_L566D	caaccgtttgaaccactaaaatttactgatgaggacgataaaccggttgag	ctcaaccggtttatcgtcctcatcagtaaattttagtggttcaaacggttg
SidH_D684A_D688A_T691D	aaatgagaaaattagtgctttaaatcaggctaaaacggatttgctggataaggaggaagaagctcgc	gcgagcttcttcctccttatccagcaaatccgttttagcctgatttaaagcactaattttctcattt

Supplementary Data 1: Identification of host interactors of SidH using quantitative proteomics

Supplementary Data 2: Identification of ubiquitination sites using mass spectrometry (sheet1- ubiquitination sites on ubiquitin; sheet2- ubiquitination sites on SidH)
