# Supplementary material for: Structural basis for the toxicity of Legionella pneumophila effector SidH
Source: Nat Commun. 2023 Nov 3;14:7068. doi: 10.1038/s41467-023-42683-8 (PMC10624908; doi:10.1038/s41467-023-42683-8)
Supplement: Supplementary file 3 — Description of Additional Supplementary Files [file 41467_2023_42683_MOESM3_ESM.pdf]

Description of Additional Supplementary Files

File Name: Supplementary Data 1
Description: Identification of host interactors of SidH using quantitative proteomics

File Name: Supplementary Data 2
Description: Identification of ubiquitination sites using mass spectrometry (sheet1- ubiquitination sites on ubiquitin; sheet2- ubiquitination sites on SidH)

File Name: Supplementary Movie 1
Description: growth of non-transfected (control) HEK cells using live microscopy

File Name: Supplementary Movie 2
Description: growth of HEK cells transfected with SidH WT using live microscopy

File Name: Supplementary Movie 3
Description: growth of HEK cells transfected with SidH (K71A, A117E, R819E) using live microscopy
