# Supplementary material for: Structural basis for the toxicity of Legionella pneumophila effector SidH
Source: Nat Commun. 2023 Nov 3;14:7068. doi: 10.1038/s41467-023-42683-8 (PMC10624908; doi:10.1038/s41467-023-42683-8)

Fig. 3E - Size Exclusion chromatography of SidH Hexa Mutant

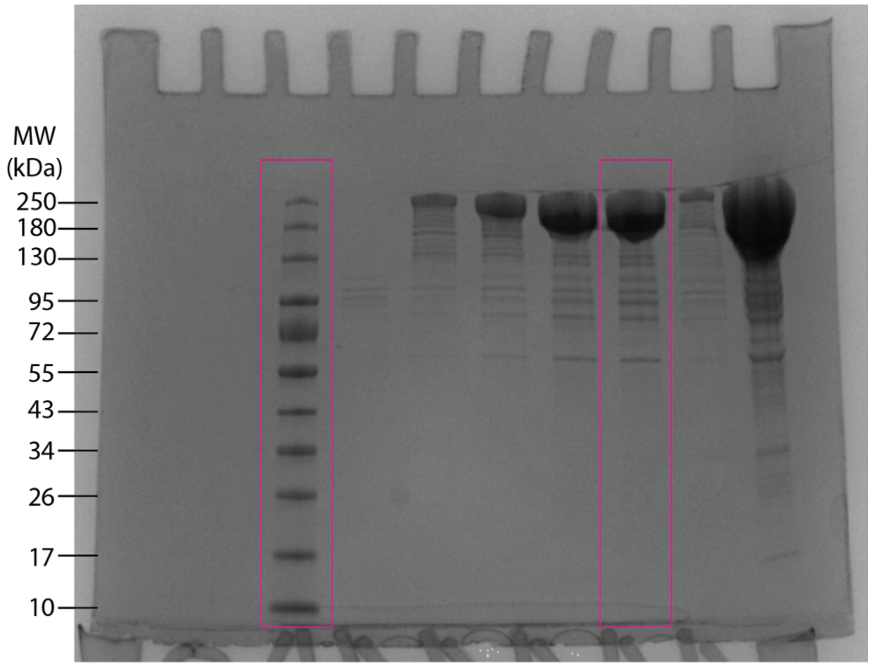

Fig. 4B - Analytical Size Exclusion chromatography

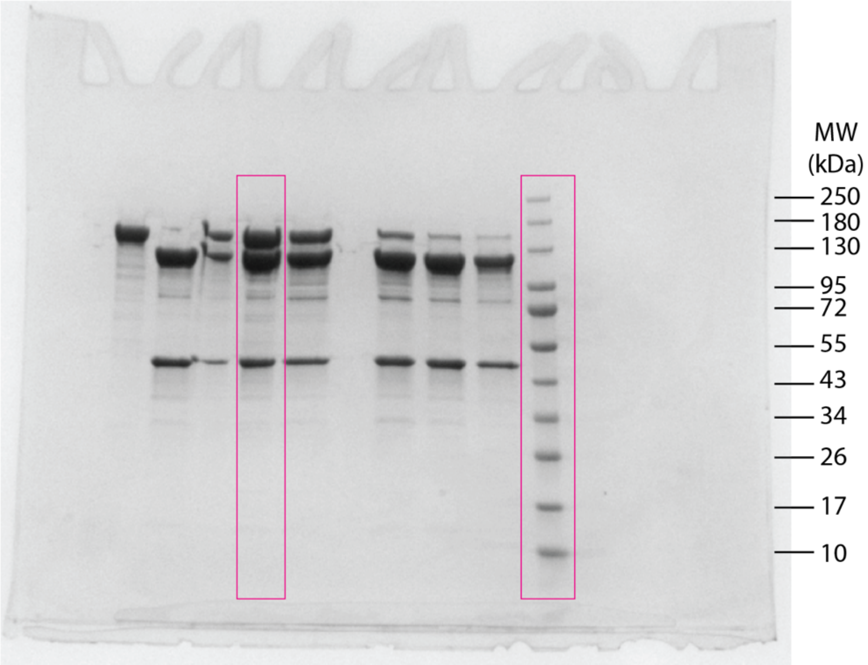

Fig. 4D - SDS-PAGE gel for Analytical Size Exclusion chromatography samples

Left upper panel

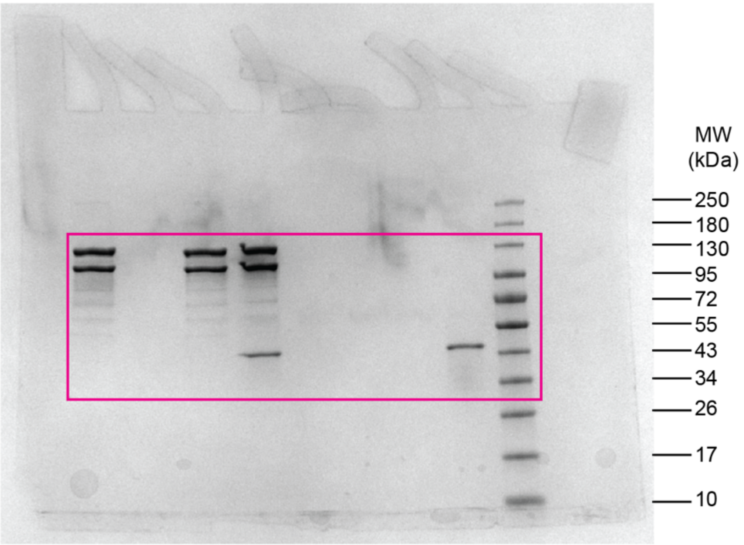

Left lower panel

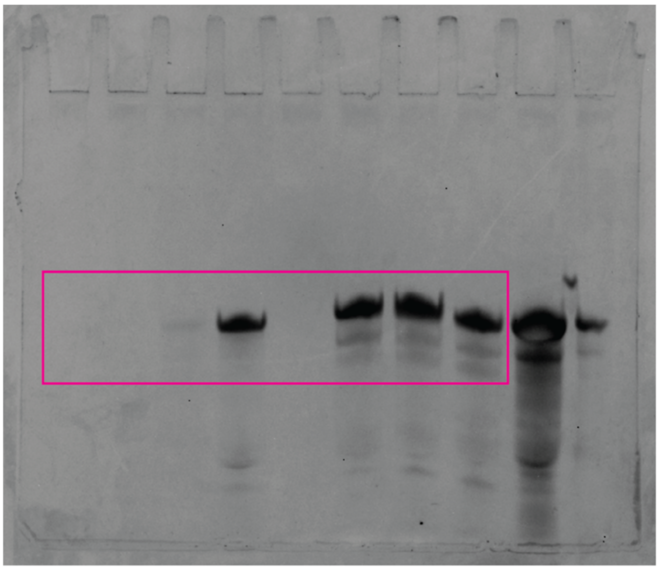

Right panel

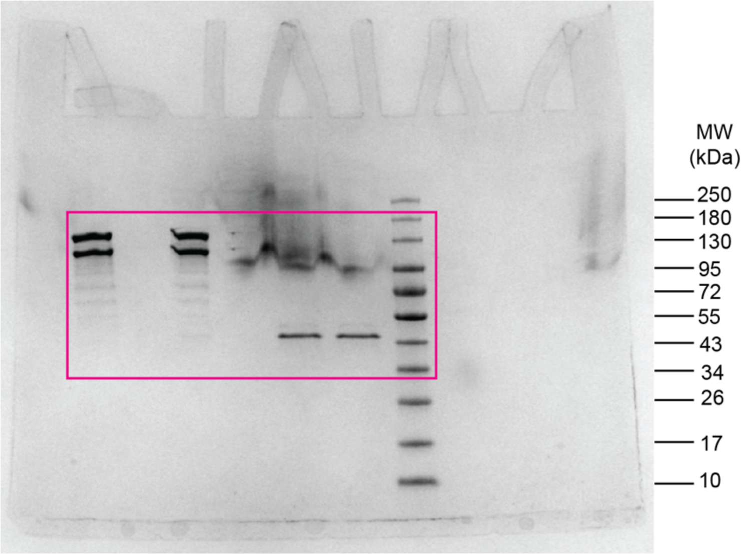

Fig. 5C - Western Blot for puromycin assay

Anti-puromycin

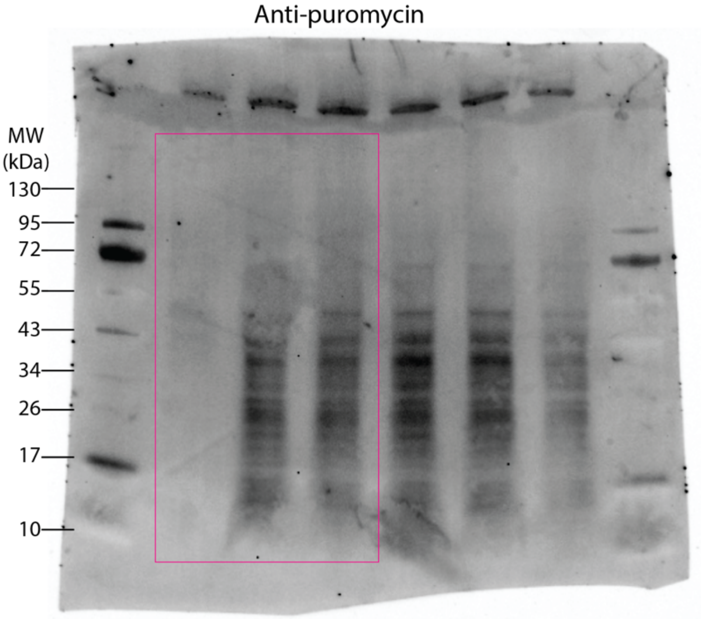

Anti-GFP

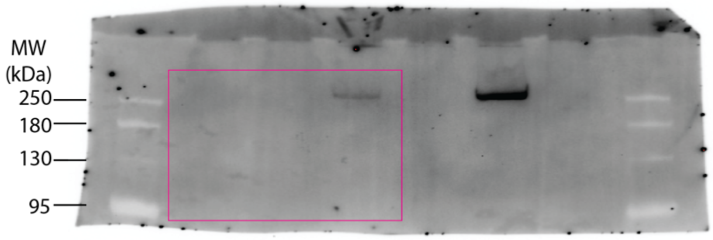

Anti-Tubulin

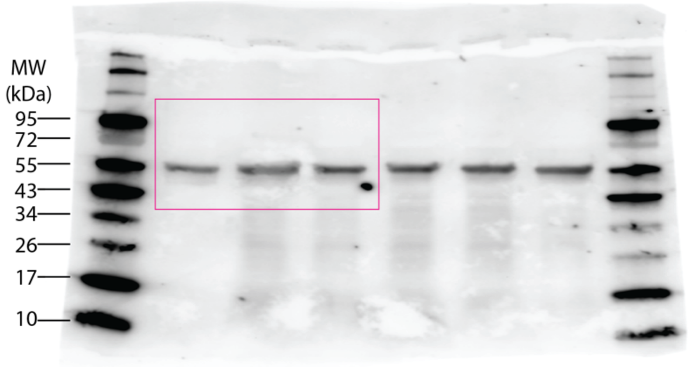

Supp. Fig. 1A - SidH FL purification

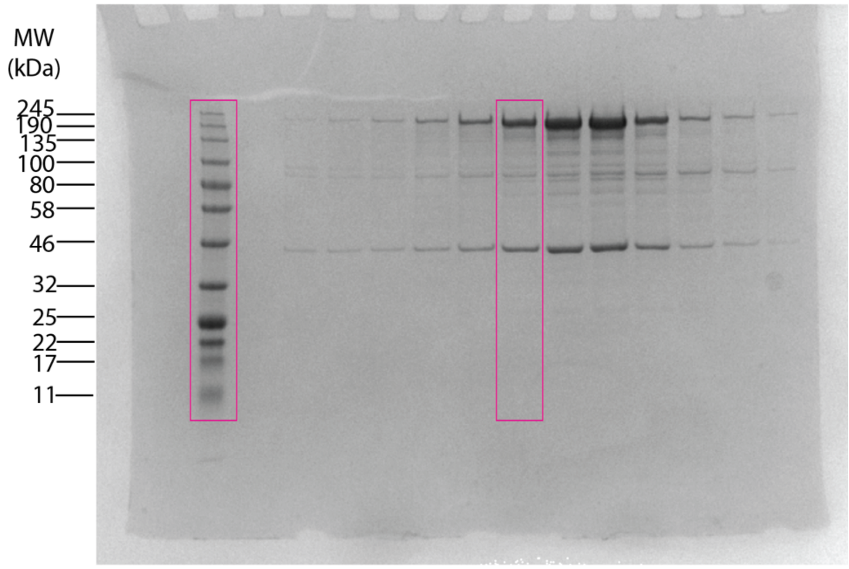

Supp. Fig. 1C

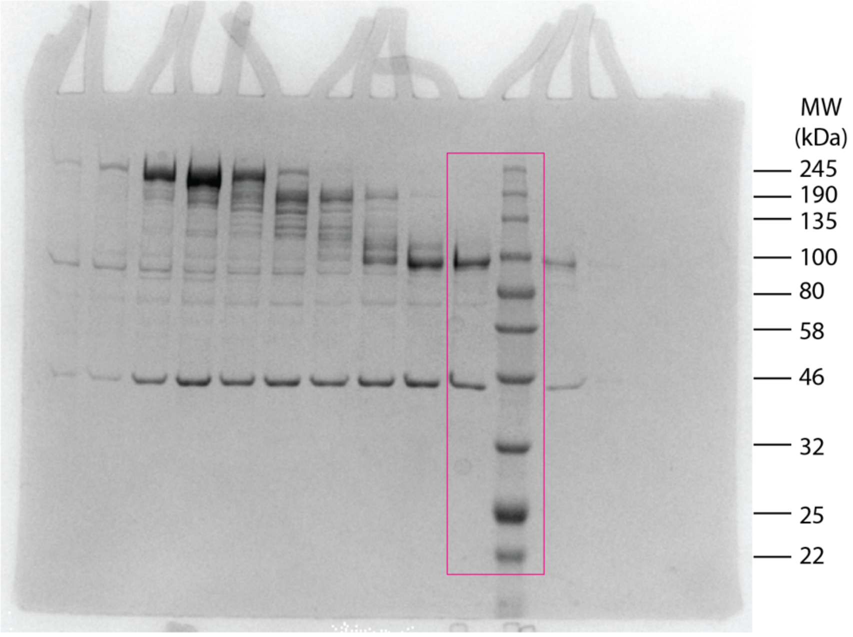

Supp. Fig. 7B - western blots for the toxicity assay

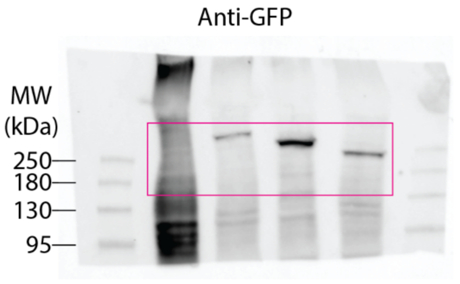

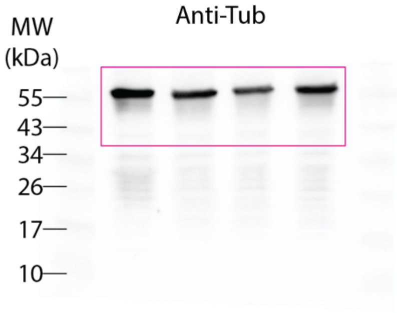

Supp. Fig. 11A

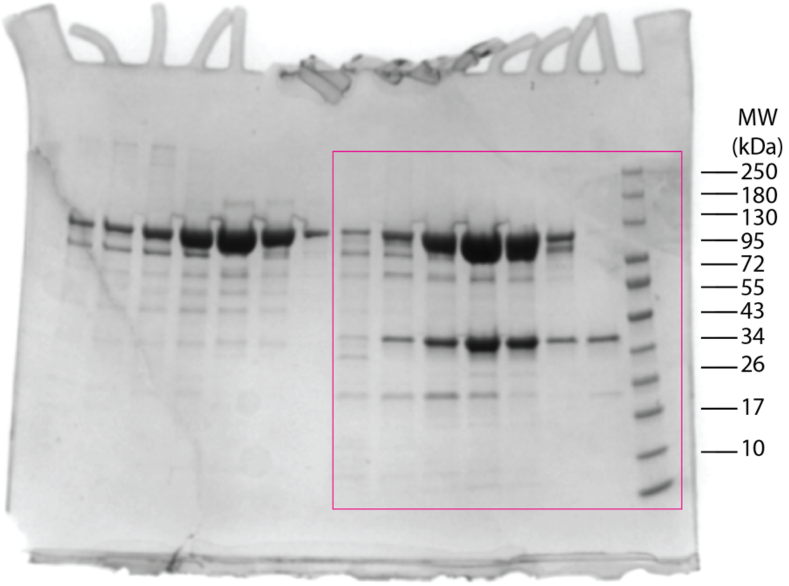

Supp. Fig. 11B

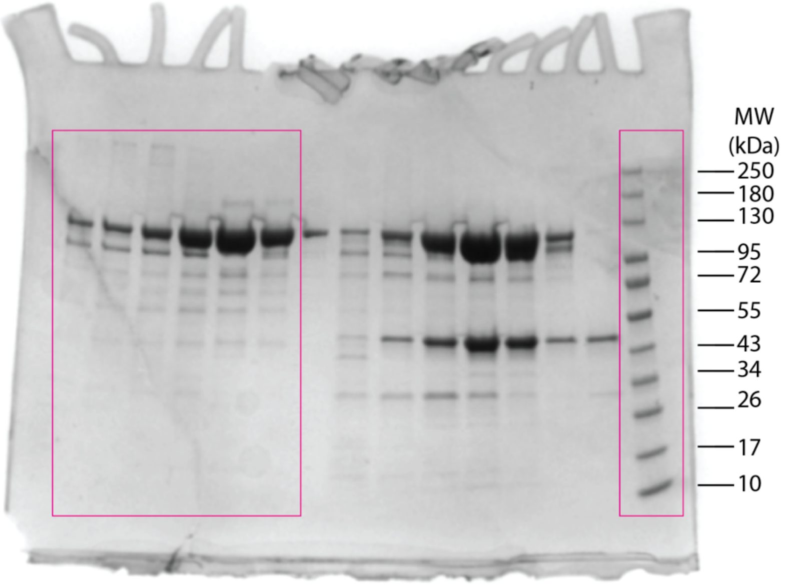

Supp. Fig. 11C

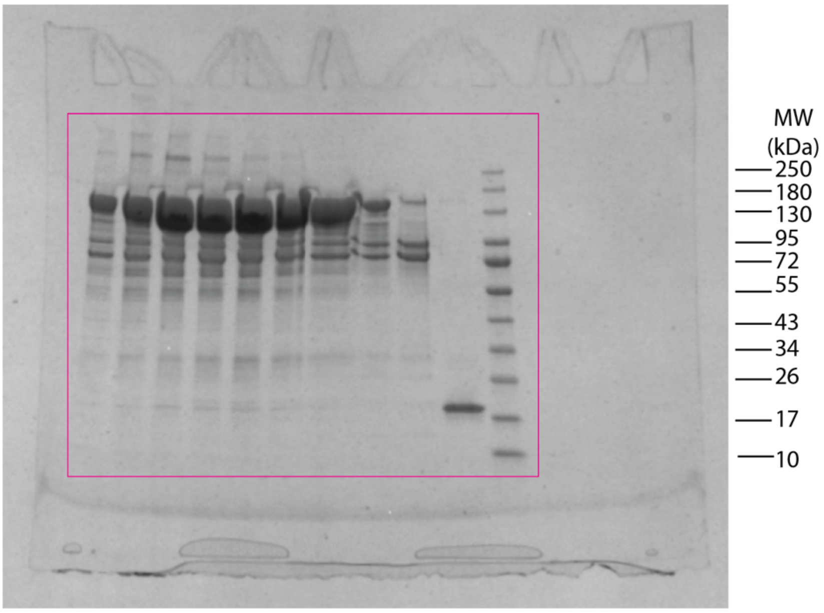

Suppl. Fig. 12A - western blots for the toxicity assay

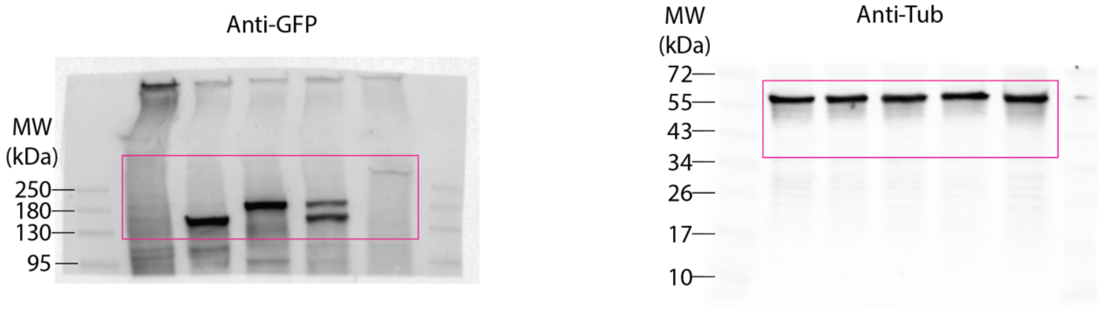

Supp. Fig. 12C - western blots for the toxicity assay

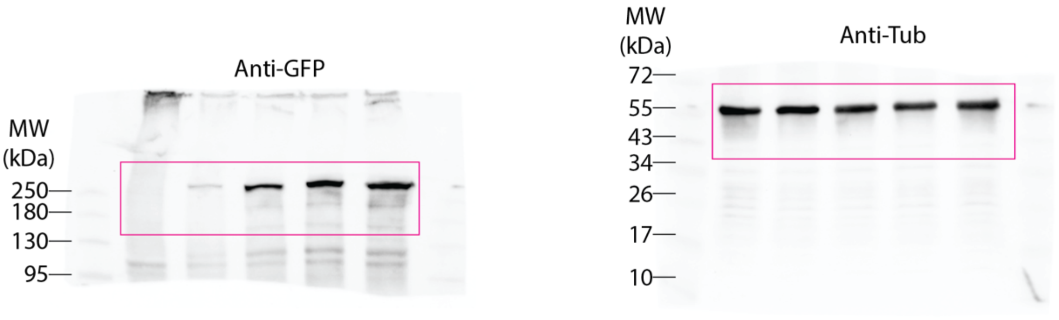

Supp. Fig. 13A

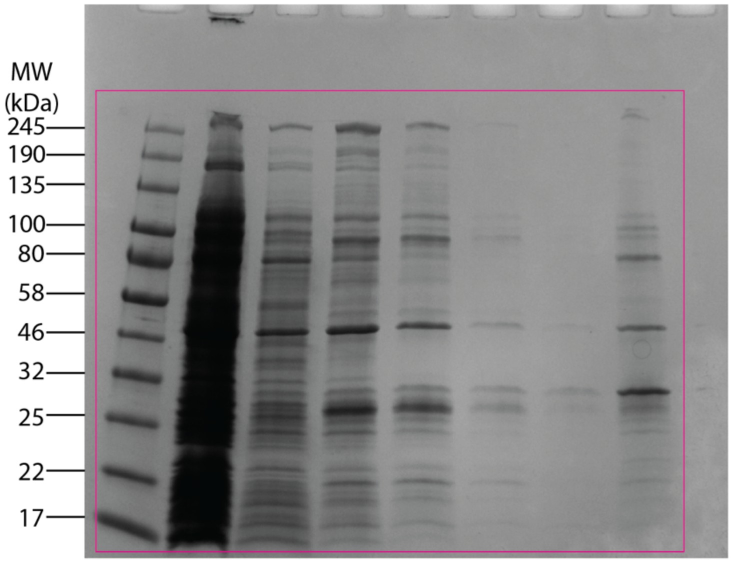

Supp. Fig. 13B

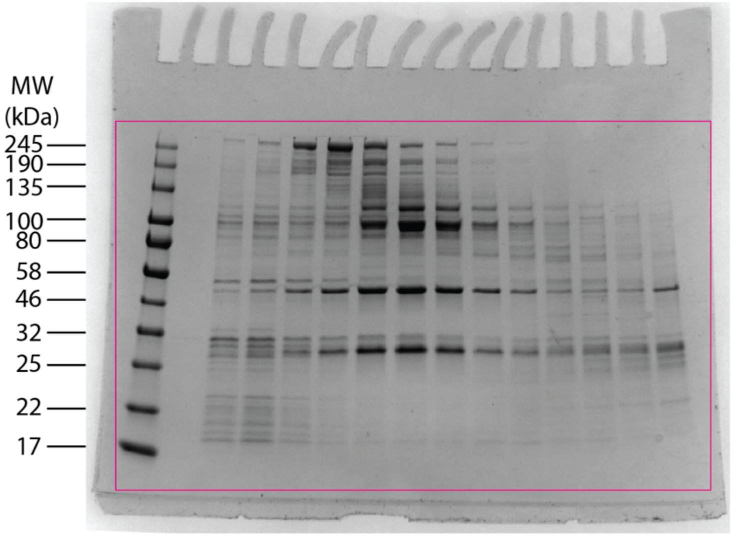

Supp. Fig. 13C

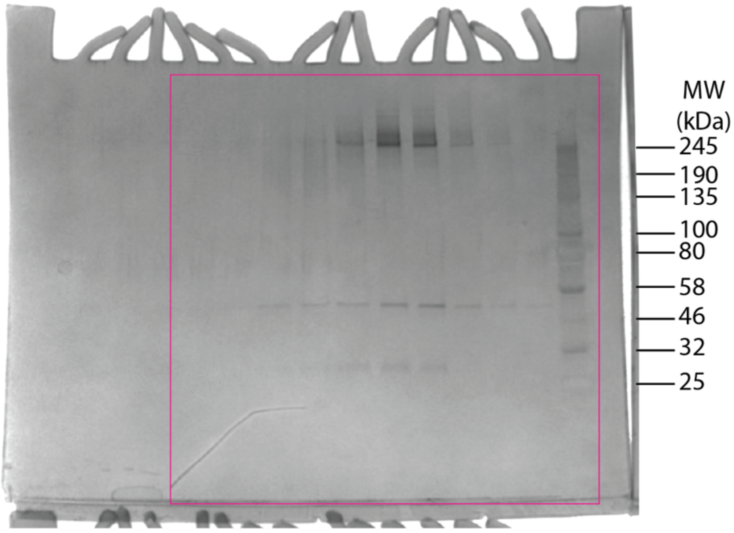

Supp. Fig. 15B - Ubiquitination assay

Top Panel

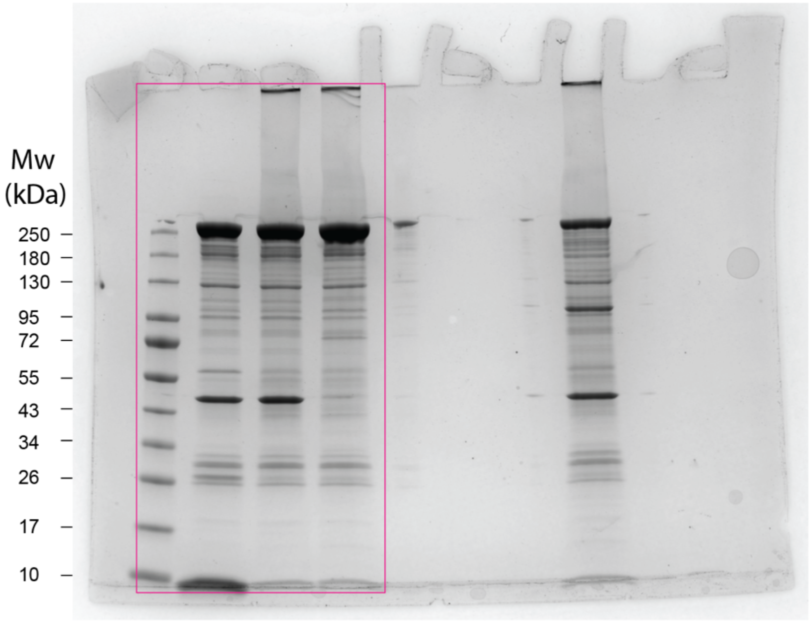

Middle Panel

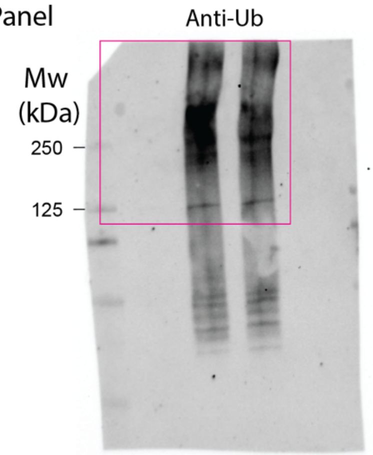

Lower Panel

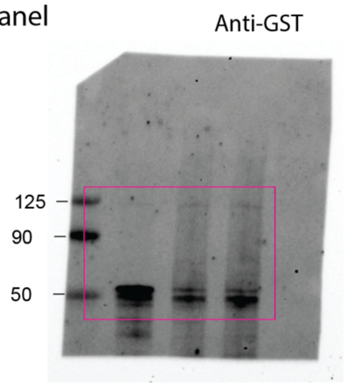

Supp. Fig. 17B, Left Panel - western blots for the toxicity assay

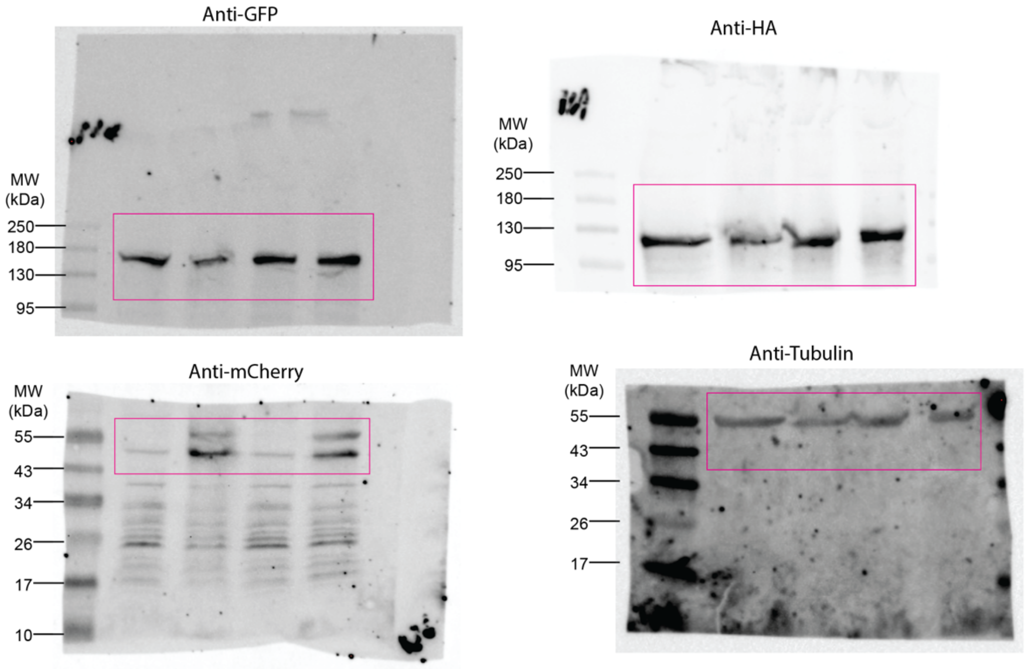

Supp. Fig. 17B, Right Panel - western blots for the toxicity assay

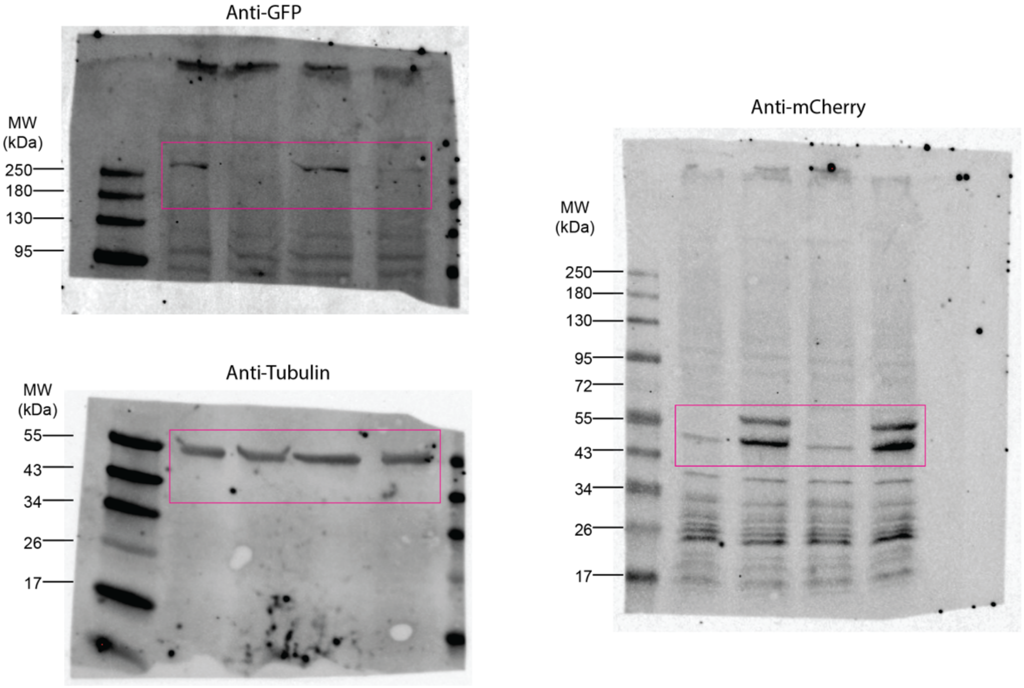

Supp. Fig. 18B - westernblot of expressed protein in infection

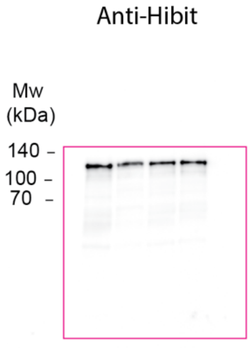

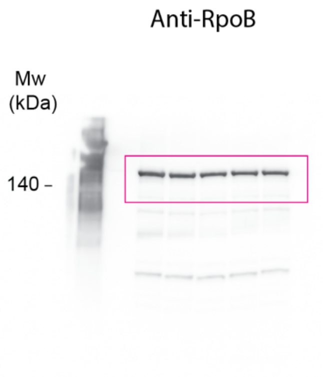

Supplement: Supplementary file 10 — Source Data [file 41467_2023_42683_MOESM10_ESM.zip › SourceData.pdf]
